# Exosomal microRNAs are novel circulating biomarkers in cigarette, waterpipe smokers, E-cigarette users and dual smokers

**DOI:** 10.1186/s12920-020-00748-3

**Published:** 2020-09-10

**Authors:** Kameshwar P. Singh, Krishna P. Maremanda, Dongmei Li, Irfan Rahman

**Affiliations:** 1 Department of Environmental Medicine, University of Rochester Medical Center, Box 850, 601 Elmwood Avenue, Rochester, NY 14642 USA; 2 Department of Clinical & Translational Research, University of Rochester Medical Center, Rochester, NY USA

**Keywords:** Plasma, Extracellular vesicles, Exosomes, microRNA, E-cig users, Waterpipe smokers, Cigarette smokers

## Abstract

**Background:**

Electronic cigarettes (e-cigs) vaping, cigarette smoke, and waterpipe tobacco smoking are associated with various cardiopulmonary diseases. microRNAs are present in higher concentration in exosomes that play an important role in various physiological and pathological functions. We hypothesized that the non-coding RNAs transcript may serve as susceptibility to disease biomarkers by smoking and vaping.

**Methods:**

Plasma exosomes/EVs from cigarette smokers, waterpipe smokers and dual smokers (cigarette and waterpipe) were characterized for their size, morphology and TEM, Nanosight and immunoblot analysis. Exosomal RNA was used for small RNA library preparation and the library was quantified using the High Sensitivity DNA Analysis on the Agilent 2100 Bioanalyzer system and sequenced using the Illumina NextSeq 500 and were converted to fastq format for mapping genes.

**Results:**

Enrichment of various non-coding RNAs that include microRNAs, tRNAs, piRNAs, snoRNAs, snRNAs, Mt-tRNAs, and other biotypes are shown in exosomes. A comprehensive differential expression analysis of miRNAs, tRNAs and piRNAs showed significant changes across different pairwise comparisons. The seven microRNAs that were common and differentially expressed of when all the smoking and vaping groups were compared with non-smokers (NS) are hsa-let-7a-5p, hsa-miR-21-5p, hsa-miR-29b-3p, hsa-let-7f-5p, hsa-miR-143-3p, hsa-miR-30a-5p and hsa-let-7i-5p. The e-cig vs. NS group has differentially expressed 5 microRNAs (hsa-miR-224-5p, hsa-miR-193b-3p, hsa-miR-30e-5p, hsa-miR-423-3p, hsa-miR-365a-3p, and hsa-miR-365b-3p), which are not expressed in other three groups. Gene set enrichment analysis of microRNAs showed significant changes in the top six enriched functions that consisted of biological pathway, biological process, molecular function, cellular component, site of expression and transcription factor in all the groups. Further, the pairwise comparison of tRNAs and piRNA in all these groups revealed significant changes in their expressions.

**Conclusions:**

Plasma exosomes of cigarette smokers, waterpipe smokers, e-cig users and dual smokers have common differential expression of microRNAs which may serve to distinguish smoking and vaping subjects from NS. Among them has-let-7a-5p has high sensitivity and specificity to distinguish NS with the rest of the users, using ROC curve analysis. These findings will pave the way for the utilizing the potential of exosomes/miRNAs as a novel theranostic agents in lung injury and disease caused by tobacco smoking and vaping.

## Background

Electronic cigarettes (E-cigs) are battery operated various kind of device, also known as electronic nicotine delivery system. These devices produce aerosolized substances by heating a liquid, which generates a large number of chemicals due to the presence of propylene glycol, vegetable glycerin, nicotine in various concentrations, flavoring agents and other additive compounds [1]. Though E-cig aerosol contains less number of toxic chemicals than cigarette smoke, but it may produce adverse health effects. E-cig aerosol contains ultra-fine particles, heavy metals, volatile organic compounds, and numerous toxic chemicals including acetaldehyde, acrolein, toluene, and formaldehyde in lower concentration than cigarette smoke [2, 3]. Recently, E-cig users are also using this device to deliver some other harmful substances, such as tetrahydrocannabinol, cannabidiol, and hash oil [4, 5]. There are several pulmonary illnesses that have been reported in users who used nicotine or cannabis extract in E-cig [4, 6]. E-cig users develop changes in lung function which manifest peripheral obstructive airway involvement [7]. E-cig use can cause oxidative stress and endothelial cell dysfunction [8, 9], and compromised innate immune response in lungs [10]. E-cig users have increased levels of biomarkers of inflammation and oxidative stress, reduced pro-resolving anti-inflammatory mediators, and endothelial dysfunction [11]. Cigarette smoke exposure causes various diseases involving changes in miRNA expressions in the pathogenesis of atherosclerosis, chronic obstructive pulmonary disease (COPD) and lung cancer [12–15]. Waterpipe tobacco smoking also causes various acute and chronic health effects including cardiovascular disease, chronic bronchitis and cancer [16, 17]. Similarly, other study has shown that waterpipe smokers may develop COPD after chronic exposure. Exosomes are extracellular vesicles membrane bound particles of 30–150 nm. These are lipid vesicles carrying various types of biological materials such as proteins, RNAs, DNAs, transcription factors, receptors, and lipids. microRNAs are non-coding molecules of 19–23 nucleotides (nt), play major role in genes regulation associated with various biological pathways [18–20]. MicroRNAs recognized as molecular markers for different diseases [21, 22]. microRNAs are present in higher concentration in whole RNAs [23]. Exosomes are also present in various body fluids and has several pathophysiological roles [24]. The exosomes act as signaling messengers by delivering various molecules to the adjacent cells and also via systemic circulation to distant cell population.

Cigarette smoke induced release of exosomes may be involved in the progression of chronic diseases [25, 26]. Also, the role of exosomes release by cigarette smoke extract have been implicated in altering cellular functions such as endothelial dysfunction and inflammatory conditions [26–30]. Transfer RNAs (transfer ribonucleic acid, tRNAs) are small molecule have multiple role in cellular functions including protein synthesis. The tRNAs play a significant role in various cellular functions such as cell signaling, proliferation, differentiation, apoptosis and metabolism, as well as in regulation of gene expression at transcription and translation levels. Additionally, they have been reported to involve in DNA damage response, viral infection, neurodegeneration and cancer [31–34]. PIWI-interacting RNA (piRNA) are 24–31 nt long and form RNA-induced silencing complex with PIWI family proteins and plays a role in stem cell division, apoptosis, epigenetic control of transposons, telomeres and translational control. The piRNA serve as gene expression regulators by inducing histone modification and DNA methylation. Given the role of tRNAs and piRNA in several important biological functions and diseases, it was thought to determine whether their levels are altered in plasma exosomes from E-cig users, waterpipe and dual smokers. To the best of our knowledge this is the first study to report the detailed and differential analysis of tRNA and piRNA in plasma exosomes from E-cig users, waterpipe, and dual smokers.

The current study is performed to identify, characterize and compare the plasma-derived exosomal microRNAs in E-cig users (E-Cig), waterpipe smokers (WPS), dual smokers (DS) and cigarette smokers (CS) with normal/non-smokers (NS). Understanding the changes in plasma-derived exosomes and their microRNA profile will help in developing biomarkers during disease progression in smokers. This study will also be useful in unraveling the molecular mechanisms of disease progression in smokers. Therefore, we did comprehensive analysis of exosomal microRNA expression profile and their biological functions in plasma samples from normal/non-smokers, E-cig users, cigarette smokers, waterpipe, and dual smokers groups.

## Methods

### Ethic statements

All protocols, procedures and subject recruitments were approved by the Institutional Review Board (IRB)/Research Subject Review Board (RSRB) committee of the University of Rochester Medical Center, Rochester, NY [11, 35]. All subjects provided written informed consent before participating in the study. The samples from non-smokers, E-cig users, waterpipe, cigarette smokers and dual smokers (cigarette and waterpipe smokers) used in this study are described previously [11, 35].

### Biochemical and molecular methods

The following methods were used by us in our recent publication and some of the methods used here are given in detail [29, 30].

### Collection of blood plasma

The venous blood (20–25 ml) was collected from cigarette smokers, waterpipe smokers, smokers (cigarette and waterpipe), Non-smokers and E-cig users.

### Plasma exosome purification

Plasma was used for exosomal isolation as per the manufacturer’s instructions (Norgen Biotek Corporation, Cat# 57400).

### Transmission electron microscopy

Transmission electron microscopy (TEM) was used to visualize exosomes as reported earlier [30].

### Nanoparticle tracking analysis (NanoSight NS300) for size and particle concentrations

NanoSight Technology NS300 was used for particle size analyses and concentration of plasma derived EVs by nanoparticle tracking analysis (NTA) as described earlier [30, 36].

### Exosomal RNA isolation

Exosomal RNA was isolated using a kit (Norgen Biotek Corporation, Cat# 58000) [20, 30].

### Small RNA library construction and sequencing

Exosomal RNA isolated was used for small RNA library using the small RNA library preparation kit (Small RNA library prep kit, cat# 63600, Norgen Biotek Corp., Canada) as reported previously [37], and quantified using High Sensitivity DNA Analysis kit on the Agilent 2100 Bioanalyzer system (Agilent Technologies, USA). Libraries were sequenced on the Illumina NextSeq 500 sequencing platform using the NextSeq 500/550 High Output kit v2 at the Norgen Biotek Corp.

### Sequence read mapping and RNA annotation

The sequence data (Illumina NextSeq 500) derived fastq format was used in the Genboree Workbench’s exceRpt small RNA-seq pipeline for mapping with human genome [38].

### Extracellular vesicle/exosome microRNA database searches

The identified miRNAs for online databases were searched using the database (http://bioinfo.life.hust.edu.cn/EVmiRNA), Vesiclepedia (http://microvesicles.org/) [39], ExoCarta (http://exocarta.org/) [40] and miRDB (http://mirdb.org/) [41], and Venn diagrams were prepared [29].

### Data and statistical analysis

The RNA-Seq fastq files were analyzed [29], and the trimmed mean of M values (TMM) method was used to normalize the summarized count data for further miRNA analysis [42]. The DESeq package of R/Bioconductor was used for miRNA differential analysis across different groups. Pairwise miRNA differences were conducted through linear contrasts within the DESeq2 model framework [43]. The false discovery rate (FDR) was accounted for at 5% using the Benjamini-Hochberg method for each pairwise comparison. A heatmap of significant miRNA selected from the pairwise comparisons was generated using the pheatmap function with the ward. D2 clustering method in statistical analysis software R [29]. A receiver operating characteristic (ROC) curve was generated using the *ROCit* package using software R (R Core Team, 2019). Pathway and gene enrichment analyses were conducted through the FunRich software [44].

### Functional over-representation analysis and gene set enrichment analysis of microRNA

Enrichment analysis and annotation tool, such as miEAA (http://ccb-compute2.cs.uni-saarland-de/mieaa_tool/; accessed in August 2019) was used to identify various microRNA expression levels and functions [45]. GeneTrail was used for miRNA set enrichment (FDR adjustment) analyses and gene ontology, pathways, and disease and over-representation analysis [29].

## Results

### Isolation and characterization of plasma-derived extracellular vesicles

**Fig. 1:**
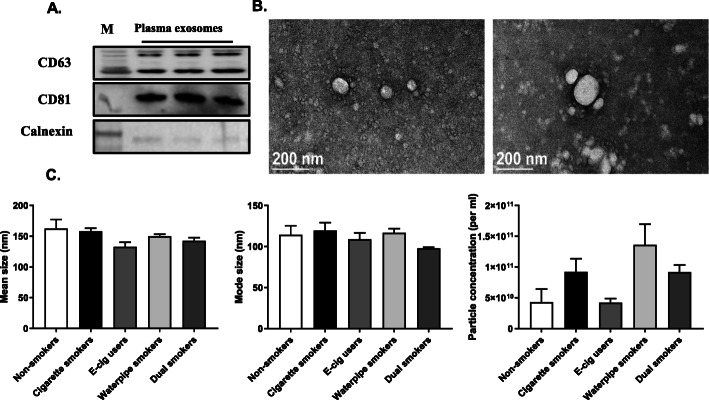
Isolation and characterization of plasma-derived EVs/Exosomes. **a** Immunoblot analysis of proteins isolated from plasma EVs. **b** Representative TEM images of plasma-derived EVs/Exosomes. **c** Particle size depicted as mean and mode, and particle concentration were estimated using NanoSight NS300 (*n* = 3/ group). Equal amount of protein was loaded as assessed by Ponceau staining. Figure 1**a** full length western blot images are available in Suppl. Fig. 1A and labeled as “original western blots”. Positive control: human lung homogenate

Plasma EVs were characterized for their size and morphology using TEM. Particles are mostly in exosomal range nonetheless large sized vesicle populations were also observed. Immunoblot analysis showed the presence of exosomal markers CD63 and CD81 and negative for calnexin and any endoplasmic reticulum contamination (Fig. 1a). Further, nanoparticle tracking analysis did not show any significant difference either in the size range or concentrations across all the groups observed (Fig. 1 b-c).

### Input read alignment and small RNA biotype mapping

**Fig. 2:**
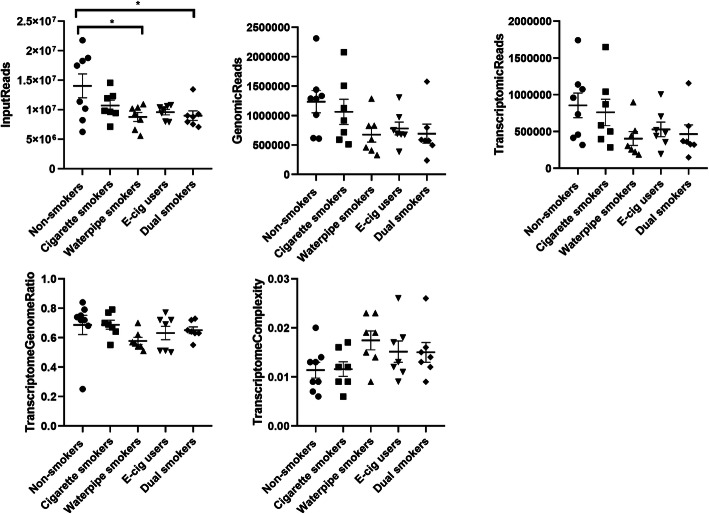
The small RNA analysis quality control results of individual samples in non-smokers, cigarette smokers, waterpipe smokers, E-cig users and dual smokers

The reading obtained from sequencing were used for alignment and mapping to the human genome after clipping and quality filtering. The input reads in plasma exosomes were between 5.58 million reads to 21.75 million reads between NS (14,031,359 ± 2,019,014), CS (10,688,617 ± 917,325), WP (8,765,589 ± 768,710), E-cig users (9,570,944 ± 455,585) and DS (8,988,900 ± 798,445). Reads were mapped to human rRNA to exclude rRNA sequences before mapping to human genome. The percentage of input reads alignment from each subject in individual group was given in Fig. 2. The input reads were significantly lower in WP and DS in comparison to NS subjects (*P* < 0.05).

**Fig. 3:**
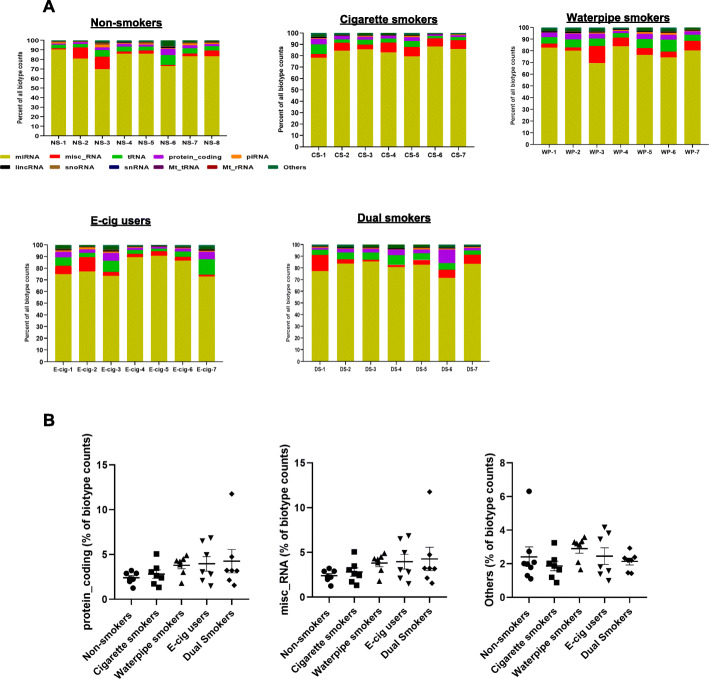
Relative biotype distribution from each sample. **a** This graph represents average percentage of biotype counts of each sample from non-smokers, cigarette, waterpipe smokers and E-cigarette smokers. **b** Comparison of relative biotype distribution of non-smokers, cigarette smokers, waterpipe smokers, E-cigarette users and dual smokers

The reads mapped to human genome were classified into various small RNA biotypes. After mapping and excluding rRNA, the microRNAs mapped along with all other RNA transcripts in GENCODE. The microRNA was between 78 and 81% of all biotype counts in all NS, CS, WP, E-cig and DS groups. There was significant lower counts of Mt-tRNA observed in CS, WP, E-cig and DS (*P* < 0.05) in comparison to NS. Further, snoRNA counts were also significantly (*P* < 0.05) lower in all groups in comparison to NS (Fig. 3 a, b).

### Comparison of microRNAs expression profiles between non-smokers, E-cig users, cigarette smokers, waterpipe smokers and dual smokers

#### Multidimensional scaling (MDS) plot

**Fig. 4:**
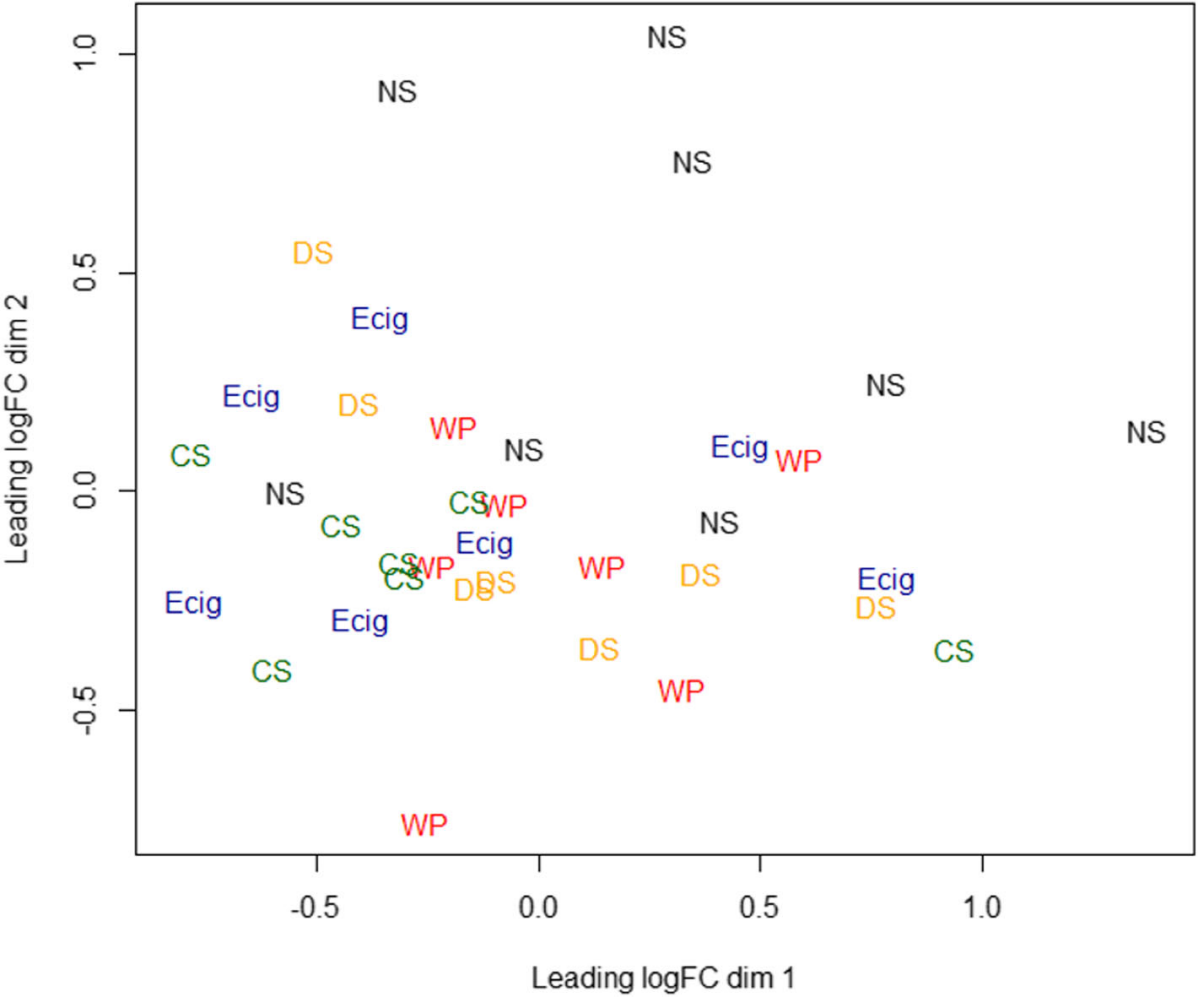
Multidimensional Scaling based on differential microRNA expression in individual samples of non-smokers, cigarette smokers, waterpipe smokers, E-cig users and dual smokers

The MDS plot was generated using the microRNAs that exhibited largest distances across all the samples from subjects of the NS, CS, WP, E-cig and DS groups. Majority of the NS samples cluster separately whereas all the smokers (CS, WP and DS), and E-cig users cluster separately suggesting these effects may be due to common tobacco use (Fig. 4).

#### Volcano plot

**Fig. 5:**
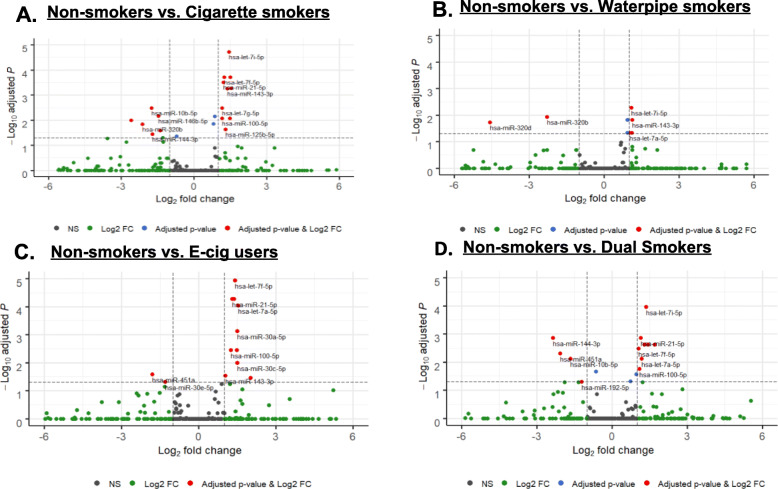
Volcano plots. Volcano plot showing the relation between *P*-values of the changes in differentially expressed microRNA, and fold change in non-smokers, cigarette, waterpipe, E-cigarette users and dual smokers. Volcano plot is useful for visualizing the magnitude of the fold changes seen between the two groups being compared. **a** Non-smokers versus cigarette smokers. **b** Non-smokers versus waterpipe smokers. **c** Non-smokers versus E-cig users. **d** Non-smokers versus dual smokers

Majority of the differentially expressed microRNA from NS vs. CS, NS vs. WP, NS vs. E-cig users, NS vs. DS (Fig. 5 a-d) and CS vs. E-cig, CS vs. WP, CS vs. DS, WP vs. DS (Suppl. Fig. 2 A-D) are presented as volcano plot. The volcano plots were made by plotting the -log_10_ of adjusted *p*-values on the y-axis, and the log_2_ fold change between two groups on the x-axis showing the up- and down regulation appearance similar distance from the center. Some microRNAs with extreme log2 fold changes were not included in the volcano plot in order to make the scales of the volcano plots consistent among different comparisons. The microRNA values plotted show two regions with highest magnitude of fold change and high statistical significance. The highlighted spots (red color) with microRNA names are with greatest difference (at least two fold changes) in expression and statistically significant (adjusted *P* < 0.05) after correction for multiple testing.

#### Hierarchical clustering of microRNAs

**Fig. 6:**
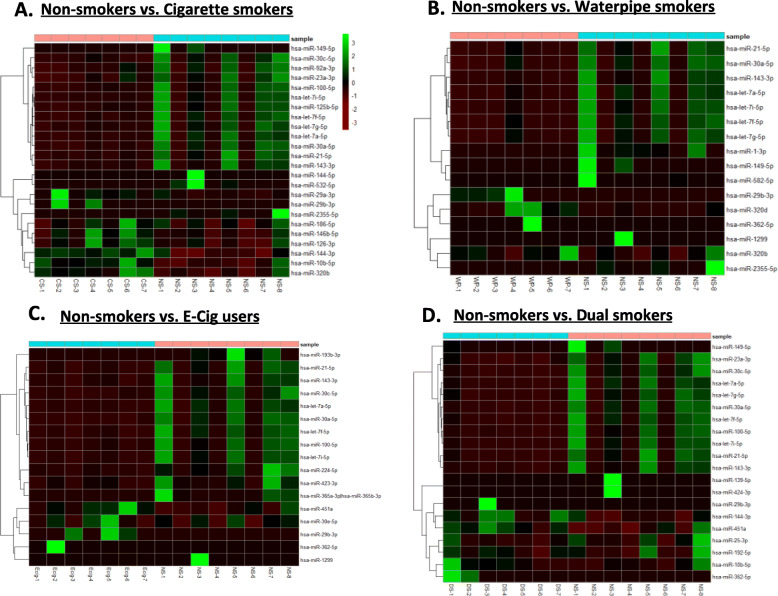
Hierarchical cluster analysis of differentially expressed miRNAs. **a** Heatmap clustering of the differentially expressed miRNAs significant among non-smokers vs. cigarette, smokers. **b** Heatmap clustering of the differentially expressed miRNAs significant among non-smokers vs. waterpipe smokers. **c** Heatmap clustering of the differentially expressed miRNAs significant among non-smokers vs. E-cigarette users. **d** Heatmap clustering of the differentially expressed miRNAs significant among non-smokers vs. dual smokers. The dendrogram shows clustering of pairwise comparisons among the different groups (non-smokers vs. cigarette smokers, non-smokers vs. waterpipe smokers, non-smokers vs. E-cigarette users and non-smokers vs. dual smokers)

The hierarchical cluster analyses of differentially expressed microRNAs were performed in all the groups. The heat maps were generated using normalized values from individual samples from NS vs. CS, NS vs. WP, NS vs. E-cig users, NS vs. DS (Fig. 6 a-d) and CS vs. E-cig, CS vs. WP, CS vs. DS, WP vs. DS (Suppl. Fig. 3 A-D). In the heatmap, each row represents individual microRNA and each column an individual sample. The microRNA clustering on the left indicates hierarchical clustering of significant microRNA. The color scale at the right side in panel A indicates the relative expression level of microRNA in all samples. The red color indicates lower level than the mean and green a level higher than the mean.

#### Differentially expressed microRNAs in E-cig versus non-smokers group

The microRNAs that were most differentially expressed in plasma exosomes of E-cig users in comparison to NS are presented in Suppl. Table 1. There were a total of 17 microRNAs that were differentially altered in E-cig users in comparison to NS. Among them 4 were downregulated and 13 were upregulated as shown in Suppl. Table 1. The upregulated microRNAs include hsa-miR-365a-3, hsa-miR-365b-3p, hsa-let-7f-5p, hsa-miR-1299, hsa-miR-21-5p, hsa-let-7i-5p, has-let-7a-5p, hsa-miR-30a-5p, hsa-miR-193b-3p, hsa-miR-100-5p, hsa-miR-423-3p, hsa-miR-30c-5p, hsa-miR-143-3p and hsa-miR-224-5p,. The downregulated expression of 4 microRNAs includes hsa-miR-362-5p, hsa-miR-29b-3p, hsa-miR-451a and hsa-miR-30e-5p.

#### Differentially expressed microRNAs in cigarette smokers versus non-smokers group

The differentially expressed microRNAs that were significantly altered between CS versus NS were given in Suppl. Table 2. A total of 24 microRNAs were significantly altered of which 16 were upregulated and 8 were downregulated as shown in Suppl. Table 2. The maximum fold change in upregulated microRNAs are hsa-mir-149-5P (20.29 fold), hsa-miR-532-5p (19.79) andhsa-miR-2355-5p (19.65), while downregulated include hsa-miR-29b-3p (23.57), hsa-miR-29a-3p (2.58), and hsa-miR-320b (− 2.13).

#### Differentially expressed microRNAs in waterpipe smokers versus non-smoker group

Sixteen miRNAs were differentially expressed in WP versus NS comparisons as shown in Suppl. Table 3. The maximum fold change observed in upregulated microRNAs are hsa-miR-2355-5p (39.81 fold), hsa-miR-149-5p (29.22) and hsa-miR-582-5p (23.15and downregulated are hsa-miR-362-5p (45.35), hsa-miR-29b-3p (24.62) and hsa-miR-320d (− 4.57).

#### Differentially expressed microRNAs in dual smokers versus non-smokers

The DS vs NS groups have shown total 20 differential expressed microRNAs that are shown in Suppl. Table 4. The top fold change upregulated microRNAs are hsa-miR-149-5p (29.29 fold), hsa-miR-139-5p (16.41), hsa-miR-424-3p (16.01), and downregulated microRNAs are hsa-miR-362-5p (44.11 fold), hsa-miR-29b-3p (21.54), and hsa-miR-144-3p (2.36).

#### Differentially expressed microRNAs in E-cig users versus cigarette smokers

Total 9 microRNAs were altered significantly in E-cig users versus CS of which 5 were upregulated and 4 were downregulated (Suppl. Table 5). Among the top fold changed microRNAs, upregulated in E-cig are hsa-miR-362-5p (19.67), hsa-miR-2355-5p (19.62), hsa-miR-532-5p (19.41), and downregulated microRNAs are hsa-miR-365a-3p (− 24.12), hsa-miR-1299 (− 24.01), and hsa-miR-193b-3p (− 7.85).

#### Differentially expressed microRNAs in waterpipe smokers versus cigarette smokers

Differentially expressed microRNAs in WP versus CS are given in Suppl. Table 6. Out of six microRNAs differentially expressed, the upregulated are hsa-miR-532-5p (21.30 fold), hsa-miR-362-5p (20.47), hsa-miR-144-5p (19.42), and downregulated are hsa-miR-1299 (− 23.53), hsa-miR-582-5p (− 22.63), hsa-miR-1-3p (− 6.72) in WP compared to CS.

#### Differentially expressed microRNAs in dual smokers versus cigarette smokers

The 5 microRNAs that were differentially expressed in DS versus CS are shown in Suppl. Table 7. hsa-miR-424-3p (20.26) is upregulated, while hsa-miR-144-5p (21.40), hsa-miR-532-5p (20.74), hsa-miR-2355-5p (19.26), and hsa-miR-362-5p (19.22) were downregulated in CS compared to DS .

#### Differentially expressed microRNAs in waterpipe smokers versus dual smokers

The 4 microRNAs differentially downregulated in WP compared to CS are presented with fold changes [hsa-miR-2355-5p (39.43), hsa-miR-1299 (21.29), hsa-miR-582-5p (21.25), hsa-miR-1-3p (7.29) and upregulated microRNAs are hsa-miR-139-5p (22.07), hsa-miR-424-3p (21.71) in Suppl. Table 8.

#### Overlap of microRNA expression in plasma exosomes

**Fig. 7:**
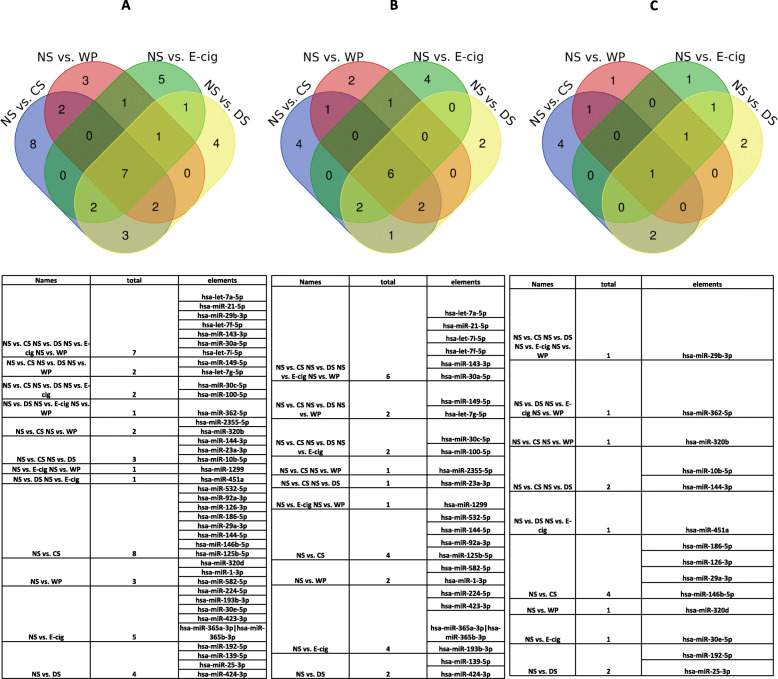
Venn diagram showing the overlap of differentially expressed microRNAs identified from comparing four groups: non-smokers vs. cigarette smokers, non-smokers vs. waterpie smokers, non-smokers vs. E-cig users and non-smokers vs. dual smokers. **a** The overlap of all differentially expressed microRNAs, **b** The overlap of up-regulated differentially up-regulated expressed microRNAs, and **c** The overlap of differentially down-regulated differentially expressed microRNAs

The overlaps of microRNAs expression between all four groups are presented as Venn diagram in Fig. 7 a-c. We have compared microRNAs expressed in all four groups: NS vs. CS, NS vs. WP, NS vs. E-cig and NS vs. DS. These groups have common expression of7 microRNAs that are hsa-let-7a-5p, hsa-miR-21-5p, hsa-miR-29b-3p, hsa-let-7f-5p, hsa-miR-143-3p, hsa-miR-30a-5pand hsa-let-7i-5p. The E-cig group has expressed 5 microRNAs (hsa-miR-224-5p, hsa-miR-193b-3p, hsa-miR-30e-5p, hsa-miR-423-3p, and hsa-miR-365a-3p|hsa-miR-365b-3p) that are specific for this group, not expressed in other three groups (Fig. 7 a). The comparison of up-regulated microRNAs (NS vs. other smoking/vaping groups) in all four groups revealed expression 6 microRNA (hsa-let-7a-5p, hsa-miR-21-5p, hsa-let-7i-5p, hsa-let-7f-5p, hsa-miR-143-3p and hsa-miR-30a-5p) common to all groups. However, there are 5 microRNA (hsa-miR-224-5p, hsa-miR-423-3p, hsa-let-7c-5p andhsa-miR-365a-3p|hsa-miR-365b-3p, and hsa-miR-193b-3p) expressed specifically to E-cig group (Fig. 7 b). When NS vs. other groups were compared for down-regulated microRNAs, hsa-mir-29b-3p was found to be common in all the groups. The microRNA expressed specifically in E-cig was hsa-mir-30e-5p and was downregulated (Fig. 7 c).

#### Receiver operating characteristic (ROC) curve for evaluation of diagnostic utility

**Fig. 8:**
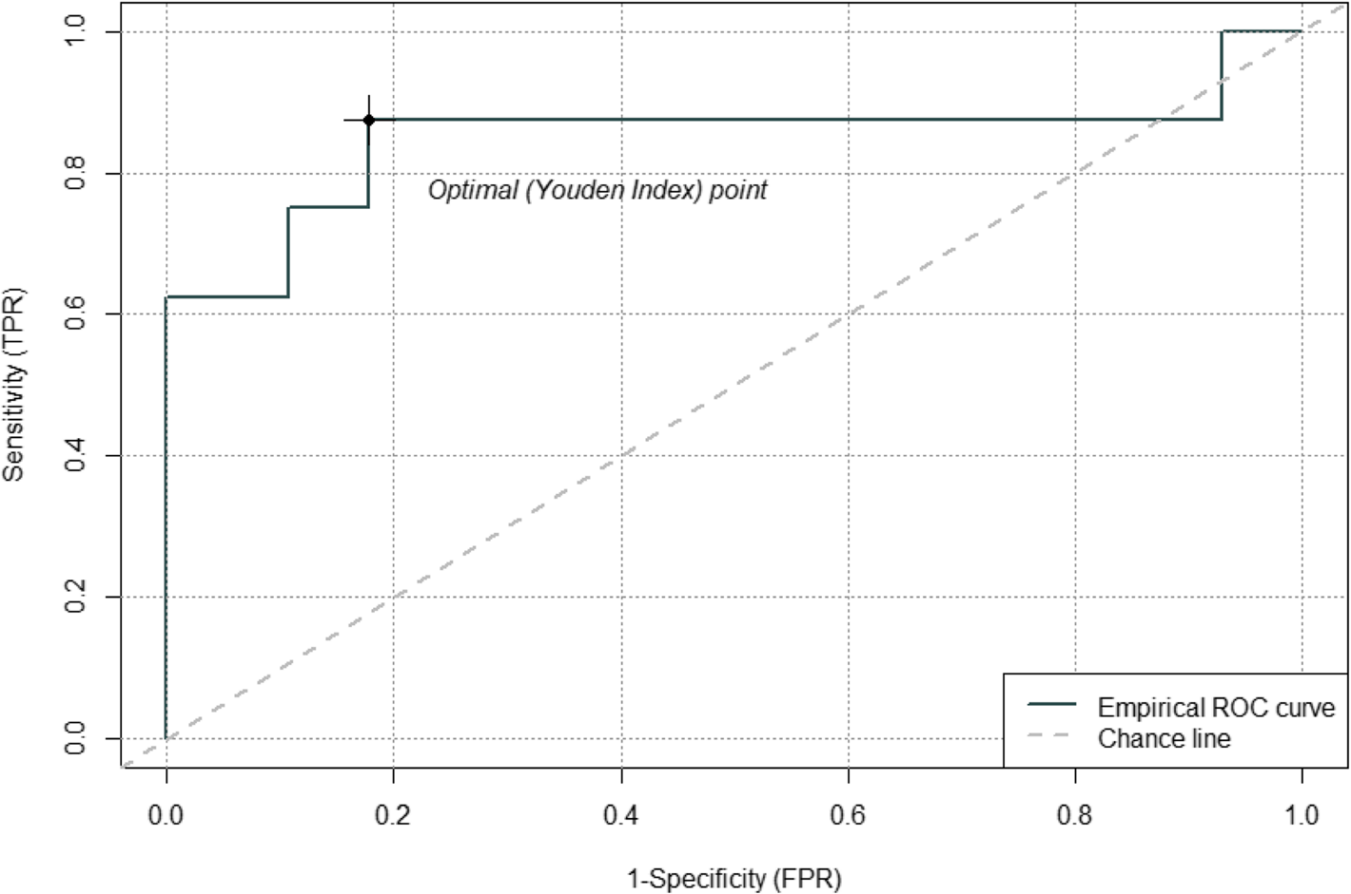
ROC curve evaluation for the diagnostic utility. ROC curve showed that both the sensitivity and specificity of the miRNA hsa-let-7a-5p marker for distinguishing normal subjects from tobacco users are greater than 80% according to the optimal Youden Index point, which captures the performance of a dichotomous diagnostic test. TRP: true positive rate; FPR: false positive rate

A ROC curve was built using one of the identified significant marker miRNA hsa-let-7a-5p to distinguish normal subjects from tobacco users (Fig. 8). The ROC curve showed that both the sensitivity and specificity of this miRNA marker are greater than 80% according to the optimal Youden Index point, which is an indicator of the performance of miRNA hsa-let-7a-5p to distinguish normal subjects from tobacco users.

#### Gene enrichment analysis of differentially expressed microRNAs

**Fig. 9:**
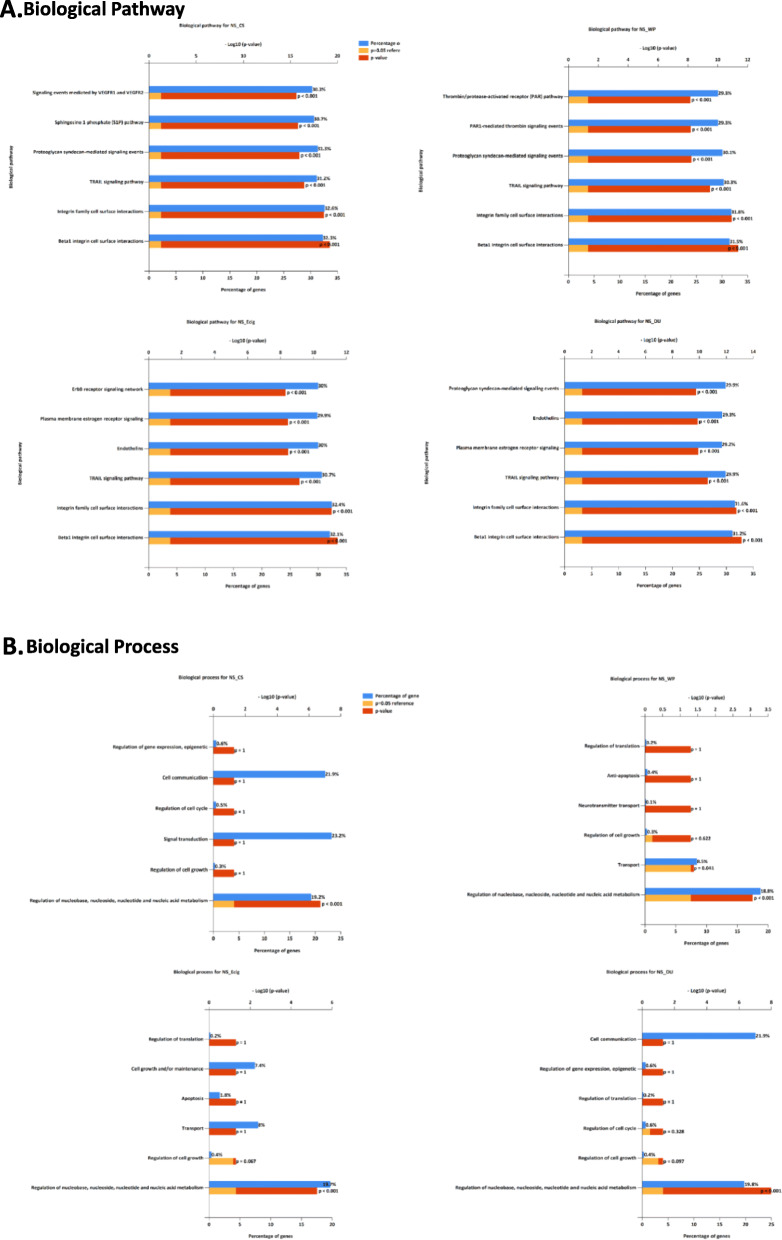

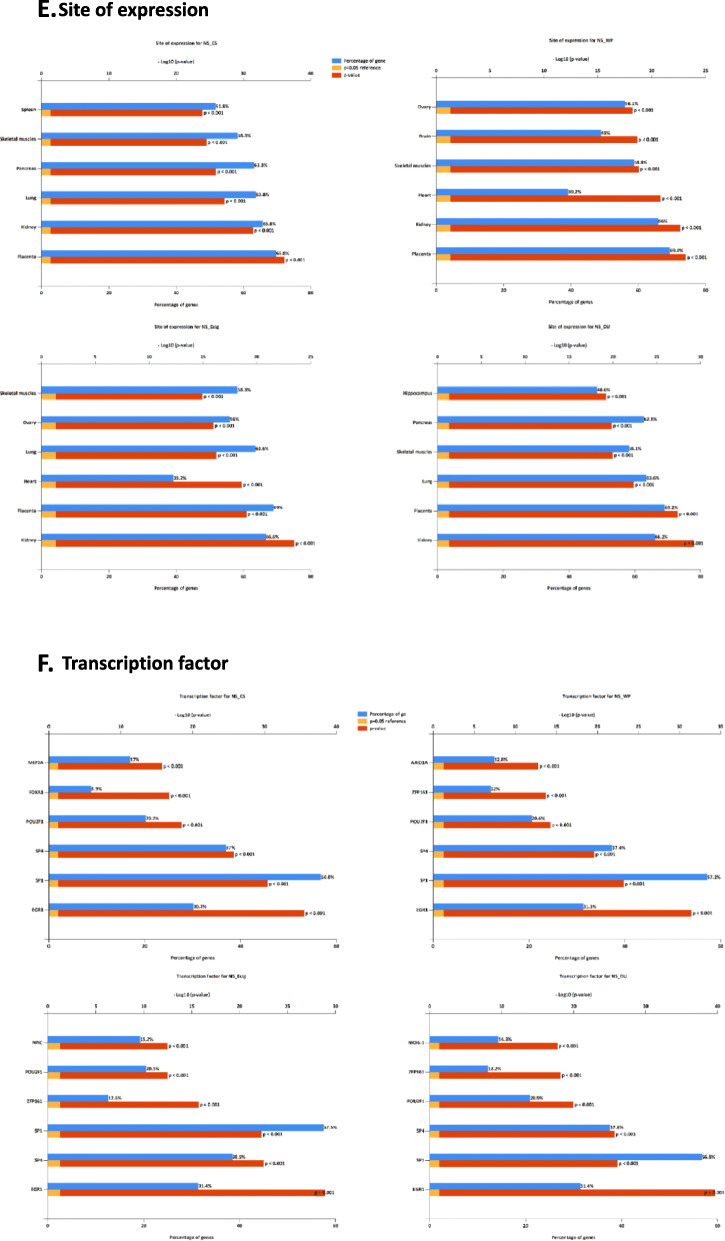
FunRich gene enrichment analysis for the differentially expressed miRNAs. Here we provide the top 6 enriched: **a** Biological process, **b** Molecular function, **c** Cellular component

The FunRich enrichment analysis of differentially expressed microRNAs was performed to explore the potential target genes in NS vs. E-cig, NS vs. CS, NS vs. WP and NS vs DS pairwise comparisons (Fig. 9a-f). The top six enriched functions with the lowest *p* values were biological pathway, biological process, molecular function, cellular component, site of expression and transcription factor in all groups. The top 3 biological pathway with the lowest *p* values were beta1 integrin cell surface interactions, integrin family cell surface interactions, and TRAIL signaling pathway common in NS vs. CS, NS vs. WP, NS vs. E-cig and NS vs. DS. The proteoglycan-mediated signaling events changed significantly in all three groups except NS vs. E-cig. In addition, endothelin biological pathways with lowest *p* values were in NS vs. E-cig and NS vs. DS.

The biological process in regulation of nucleobase, nucleoside, nucleotide and nucleic acid metabolism with lowest p value were present in all four groups. The two molecular functions with highly significant values related to transcription factor activity and extracellular matrix structural constituent were common in all four groups. The top two cellular components related to nucleus and cytoplasm were common in all four groups. The three site of expression of microRNAs with lowest p values were kidney, placenta and skeletal muscle. However, the other sites of expression related to lung with significant p values were in NS vs. CS, NS vs. E-cig and NS vs. DS. The transcription factor related EGR1, SP1, SP4 and POU2F1 were highly significant in all four groups while ZFP161 in only NS vs. E-cig and NS vs. DS. Further, pairwise comparisons were done in between E-cig vs. CS, WP vs. CS, DS vs. CS and WP vs. DS groups were presented in Suppl. Fig. 4 A-F.

#### Target genes of plasma exosomal microRNA changes

The list of target genes of plasma exosomal microRNA changes observed in NS vs. E-cig, NS vs. CS, NS vs. WP, NS vs DS and CS vs. E-cig, CS vs. WP, CS vs. DS, WP vs. DS are presented in Suppl. Table 9.

#### Differential tRNA fragments identified in plasma exosomes

The changes in tRNAs were calculated based on the trimmed mean of M values (TMM) method using normalized tRNA counts in all NS, CS, WP, E-cig and DS groups. The read counts normalized and subjected to differential expression analysis by DESeq2, to get the change in tRNA expression between different groups by pairwise comparisons. The read raw count data analysis showed 25 different types of tRNAs in plasma exosomes of all groups NS vs. CS, NS vs. WP, NS vs. E-cig and NS vs. DS. The pairwise comparison data revealed significant changes in 7 tRNAs in all NS vs. CS, NS vs. WP and NS vs. E-cig groups. However, NS vs. DS group showed changes in eight tRNAs. All four groups have significant increase in six tRNAs (tRNA^Val^, tRNA^Glu^, tRNA^Asp^, tRNA^Gly^, tRNA^Arg^ and tRNA^His^) and decrease in tRNA^Cys^. In addition, NS vs. DS group also showed significant increase in tRNA^Ile^ (Suppl. Tables 10, 11, 12, and 13). There was no significant change observed in tRNAs counts (total 24 raw counts in each) in all CS vs. DS, CS vs. WP, CS vs. E-cig and WP vs. DS.

The MDS plots were generated using the tRNAs that exhibited largest distance across all the samples from subjects of the NS, CS, WP, E-cig and DU groups. Majority of the samples from all the groups (NS, CS, WP, DS and E-cig) did not cluster together (Suppl. Fig. 5).

The differentially expressed tRNAs from NS vs. CS, NS vs. WP, NS vs. E-cig users, and NS vs. DS (Suppl. Fig. 6 A-D) are presented as volcano plot. The *p*-values on the y-axis, and the fold change between two groups on the x-axis are plotted showing the up- and down regulation appearance similar distance from the center. The tRNAs values plotted show two regions with highest magnitude of fold change and high statistical significance. The highlighted spots (red color) with tRNAs names are with greatest difference in expression and statistically significant (*P* < 0.05) after correction for multiple measurement.

The hierarchical cluster analyses of differentially expressed tRNAs were done in all different groups (Suppl. Fig. 7). The heat maps were generated using normalized values from individual sample from NS vs. CS, NS vs. WP, NS vs. E-cig users, NS vs. DS (Fig. 6 a-d). In the heat map, each row represents individual tRNA and each column individual sample. The tRNA clustering on the left indicates hierarchical clustering of significant tRNA. The color scale at the right side indicates the relative expression level of tRNA in all samples. The red color indicates lower level than the mean and green a level higher than the mean.

The overlap of tRNAs expression between all four groups are presented as Venn diagram in Suppl. Fig. 8. We have compared tRNAs expressed in all four groups: NS vs. CS, NS vs. WP, NS vs. E-cig and NS vs. DS. All the four groups have common changes in 7 tRNAs (tRNA^Val^, tRNA^Glu^, tRNA^Asp^, tRNA^Gly^, tRNA^Arg^ and tRNA^His^, and tRNA^Cys^). However, change in tRNA^Ile^ was only observed in NS vs. DS.

#### Differential expression of piRNA in plasma exosomes

Read counts of piRNAs from NS, CS, WP, E-cig, and DS groups were TMM-normalized and normalized counts were used to generate a MDS plot (Suppl. Fig. 9). There was no close clustering observed between individual samples of these groups. Normalized counts were also were processed for differential expression analysis by DESeq2 to get the data of different group pairwise comparisons.

The hierarchical cluster analyses of differentially expressed piRNAs were done in all different groups. The heatmaps were generated using normalized values from individual sample from NS vs. CS, NS vs. WP, NS vs. E-cig users, NS vs. DS (Suppl. Fig. 9 A-D). In the heat map, each row represents individual piRNA and each column individual sample. The piRNA clustering on the left indicates hierarchical clustering of significant piRNA (Suppl. Fig. 10E-H). The color scale at the right side in indicates the relative expression level of piRNA in all samples. Venn diagram showing piRNA for different groups is given in Suppl. Fig. 11). The red color indicates lower level than the mean and green a level higher than the mean. The significant piRNAs across all the pairwise comparisons are given in Suppl. Table 14.

#### Overall sequencing and metadata

The above described sequence data in fastq format with associated metadata generated from current study as described above have been deposited into the NCBI Sequence Read Archive (SRA) public repository. The deposited BioProject database with associated URL links (See Availability of Data and Materials) are described in Suppl. Tables 15, 16, 17.

## Discussion

Exosomes are secreted into biological fluids, and communicate with cells by transferring exosomal content including microRNAs to the recipient cells, thereby mediate several cellular and biological processes [37, 46, 47]. In addition, exosome releases micoRNAs during airways lung injury that involve in the progression of lung diseases [29, 47].

In this study, we have investigated the small RNA profile of plasma samples of NS, E-cig users, WP and DS using high-throughput sequencing. We have presented a comprehensive microRNAs profile of plasma exosomes in all the four groups in comparison to the NS. Our results showed that some microRNAs are common in all tobacco/E-cig users. Interestingly, we also found group specific microRNAs in different groups.

To perform a comprehensive analysis of small RNA species, we analyzed our data for several key abundant RNA biotypes. The input reads were lower in all groups, but the significant lower were seen in WP and DS. Several biotypes of known biological importance were identified, such as piRNA, tRNA, snoRNA, snRNA, lincRNA, Mt_tRNA, Mt_rRNA and microRNAs. The microRNA was most diverse RNA biotype found in comparison to all biotypes in samples from all groups. Biotypes Mt_tRNA and snoRNA were significantly lower in all groups in comparison to NS, suggesting nicotine-specific effect. Recent studies of small RNA sequence analysis of exosomes in various biofluids from healthy donors, smokers, and COPD patients reported showing changes in relative distribution of biotypes, such as microRNA, tRNA, Mt_tRNA, and snoRNA [29, 37]. We have performed further analysis of tRNA and piRNA in all NS, CS, E-cig, WP and DS groups, showing a differential regulation of these transcripts.

There are several studies related to expression of coding and non-coding RNA in CS exposed cellular models and COPD patients [48–50]. The studies related to transcriptome profiling of gene expression in E-cig vapor and CS exposed human bronchial epithelial cells and nasal epithelial cells from E-cig users [29, 51, 52]. Recently, microRNA expression profiling of E-cig exposed human airway epithelial cells has been reported [53]. The waterpipe smokers have differential gene expression in small airway epithelial cells and alveolar macrophages in comparison to non-smokers [54]. Further, waterpipe smokers small airway epithelial cells have epigenetic related changes in gene expression [55]. However, the studies related to plasma exosomal microRNA in E-cig users and WP are still unknown.

In plasma exosomes of E-cig users, we identified 17 microRNA dysregulated significantly, of which four were upregulated and 13 microRNA were downregulated in comparison to non-users/non-smokers (Suppl. Table 1). The three downregulated microRNAs in E-cig group (more than two-fold change; has-miR-365a-3p/has-miR-365b-3p, hsa-miR-1299 and has-miR-193b-3p) were associated with cancer and cardiovascular complications. The upregulated expression of microRNAs in E-cig users: hsa-miR-362-5p, hsa-miR-29b-3p, hsa-miR-451a, play role in human natural killer cell function, epithelial-mesenchymal transition, and suppression of cell migration and invasion in cancer [56–59].

The top three downregulated microRNAs in WP are hsa-miR-2355-5p, has-miR-149-5p and hsa-miR-582-5pand upregulated are hsa-miR-362-5p, hsa-miR-29b-3p and hsa-miR-320d. The microRNA hsa-miR-2355-5p is upregulated in endothelial colony-forming cell, the compromise function of this cell may lead to onset of cardiovascular disorder [60]. hsa-miR-1299 which is downregulated in E-cig users was suggested as diagnostic marker for rheumatic heart disease [61]. The two miRNAs i.e. has-miR-362-5p and has-miR-29b-3p were also upregulated similarly in E-cig users. The downregulated hsa-miR-320b was suggested in predicting reduced survival of COPD patients and hsa-miR-320d as biomarker for aortic dissection [62, 63].

We examined the differential expression of microRNA in cigarette smokers in comparison to non-smokers. A maximum number of total 24 microRNAs were differentially expressed in cigarette smokers (Suppl. Table 2). The top three downregulated microRNAs are hsa-miR-149-5p, hsa-miR-532-5p, hsa-miR-2355-5p, and upregulated hsa-miR-29b-3p, hsa-miR-150.5p, hsa-miR-29a-3p. hsa-miR-2355-5p is also upregulated in waterpipe smokers and hsa-miR-29b-3p upregulated similarly in E-cig users and waterpipe smokers. The downregulation of microRNA hsa-miR-29a-3p and hsa-miR-150-5p, and hsa-miR-29a-3p are associated with radiation therapy and fibrosis in human heart, lung and kidney [64].

The dual smokers differentially expressed total 20 microRNAs of which 13 were downregulated and 7 were upregulated (Suppl. Table 4). The top three downregulated based on fold change were hsa-miR-149-5p, hsa-miR-424-3p, hsa-miR139-5p, and upregulated were hsa-miR-362-5p, hsa-miR-29b-3p, hsa-miR-144-3p. The hsa-miR-362-5p upregulated in WP, and hsa-miR-29b-3p upregulated in both WP and E-cig users. The upregulated hsa-miR149-5p is also downregulated similarly in cigarette smokers. Downregulated has-miR-144-3p is associated with progression of lung adenocarcinoma [65].

To our surprise, when we analyzed the overlap of microRNAs in all four groups, there were 6 downregulated miRNAs (hsa-let-7a-5p, hsa-miR-21-5p, hsa-let-7i-5p, hsa-let-7f-5p, hsa-miR-143-3p and hsa-miR-30a-5p) common to all the groups. The microRNAs hsa-let-7a-5p and hsa-let-7f-5p are involved in NSCLC and typical and atypical carcinoid tumors of the lung, respectively [66, 67]. A ROC curve evaluation of diagnostic utility using miRNA hsa-let-7a-5p to distinguish normal subjects from tobacco users showed high sensitivity and specificity (greater than 80%). In addition, miRNA hsa-let-7a-5p was also found to be an identifier for the cigarette smoke treatments in lung cells [29]. This supports the validity and the usage of this miRNA as a possible theranostic marker in the future. Further, exosomal microRNA-21 derived from bronchial epithelial cells play a role in myofibroblast differentiation in COPD by cigarette smoking [68]. Further, when groups compared for upregulated microRNAs, the only microRNA has-miR-29b-3p was common in all groups (Fig. 7 c) which is involved in epithelial-mesenchymal transition [57]. As changes in these microRNAs expression are common in all the groups and may reflect tobacco-specific effect. There are four downregulated microRNAs (hsa-miR-224-5p, hsa-miR-423-3p, hsa-miR-365a-3p|hsa-miR-365b-3p, and hsa-miR-193b-3p), and one upregulated hsa-miR-30e-5p expressed specifically to E-cig group (Fig. 7 b, c). These microRNAs may be specific biomarkers for E-cig users.

Next, we performed FunRich functional enrichment analysis of differentially expressed microRNAs to explore the potential target genes in NS vs. E-cig, NS vs. CS, NS vs. WP and NS vs DS pairwise comparisons. The top six enriched functions with the lowest *p* values were biological pathway, biological process, molecular function, cellular component, site of expression and transcription factor in all groups. The top 3 biological pathway with the lowest p values were beta1 integrin cell surface interactions, integrin family cell surface interactions, TRAIL signaling pathway common in NS vs. CS, NS vs. WP, NS vs. E-cig and NS vs. DS. The proteoglycan-mediated signaling events changed significantly in all three groups except NS vs. E-cig. In addition, endothelin biological pathways with lowest p values were in NS vs. E-cig and NS vs. DS.

The biological process of regulation of nucleobase, nucleoside, nucleotide and nucleic acid metabolism with lowest p value were in all four groups. The two molecular functions with highly significant values related to transcription factor activity and extracellular matrix structural constituent were common in all four groups. The top two cellular components related to nucleus and cytoplasm was common in all four groups. The three site of expression of microRNAs with lowest p values were kidney, placenta and skeletal muscle. However, the other sites of expression related to lung with significant p values were in NS vs. CS, NS vs. E-cig and NS vs. DS. The transcription factor related EGR1, SP1, SP4 and POU2F1 were highly significant in all four groups while ZFP161 in only NS vs. E-cig and NS vs. DS. Our analysis revealed that many vital biological functions associated genes are potential target of selective plasma exosomal microRNAs in all four groups, which may be associated with lung pathophysiological conditions.

To determine the target genes of differentially expressed microRNAs in different groups, we identified 3244 (NS vs. CS), 2428 (NS vs. WP), 2223 (NS vs. E-cig users), 2887 (NS vs. DS), 538 (CS vs. WP), 784 (CS vs. E-cig users), 111 (CS vs. DS and 532 (DS vs. WP) and presented in Suppl. Table 9. The large numbers of genes are the target of differentially expressed microRNAs in all the groups suggesting several compromised biological functions in these groups.

Studies have shown that tRNA and piRNA are associated with several human diseases. Our differential expression analysis showed 25 different types of tRNAs in plasma exosomes of all groups NS vs. CS, NS vs. WP, NS vs. E-cig and NS vs. DS. The pairwise comparison data revealed significant changes in 7 tRNAs in all NS vs. CS, NS vs. WP and NS vs. E-cig groups. However, NS vs. DS group showed changes in 8 tRNAs. All four groups have significant increase in 6 tRNAs (tRNA^Val^, tRNA^Glu^, tRNA^Asp^, tRNA^Gly^, tRNA^Arg^ and tRNA^His^) and decrease in tRNA^Cys^. In addition, NS vs. DS group also showed significant increase in tRNA^Ile^ (Suppl. Tables 10, 11, 12, 13). There is no information available regarding the tRNA expression in plasma exosomes of E-cig users, WP, and DS. A recent study from our lab has shown that five tRNAs differentially expressed (tRNA^Lys^, tRNA^Gly^, tRNA^Tyr^, tRNA^Glu^and tRNA) in non-smokers vs COPD and five tRNAs (tRNA^Gly^, tRNA^Tyr^, tRNA^Leu^, tRNA and tRNA^Met^) in smoker vs. COPD pairwise comparison among the 25 enriched tRNAs [29]. tRNAs plays important role in tumor development and associated with the pathological characteristics of lung adenocarcinoma and cancer-specific survival [69]. The significant differential expression of piRNA in plasma exosomes of NS vs. CS (piR-004153, pir-019825), NS vs. WP (piR-004153, piR-019825, piR-000552, piR-014620, and piR-020450), NS vs. E-cig (piR-016658, piR-016659, piR-019825, piR-000552, and piR-017591), and NS vs. DS (piR-020365, piR-000552, and piR-017591) by pairwise comparisons were found. Further, pairwise comparison of piRNA expression in CS vs. WP (piR-000552 and piR-020450), CS vs. E-cig (piR-000552), CS vs. DS (piR-000552), and WP vs. DS (piR-019825, piR-014620, and piR-020450) also showed significant changes (Suppl. Table 14). piRNA form RNA-induced silencing complex with PIWI family proteins and plays a role in stem cell division, apoptosis, epigenetic control of transposons, telomeres and translational control. There is no study as such to study the differential expression of piRNAs in plasma exosomes of WP, E-cig users, and DS. A differential expression of piRNA-004153 was observed in non-smokers vs. smokers, similar to our finding in NS vs. CS [29].

We mapped input reads to human genome and classified to the various small RNA biotypes. To our surprise, significantly lower counts of Mt-tRNA and small nucleolar RNA were observed in CS, WP, E-cig and DS (*P* < 0.05) in comparison to NS (Fig. 3 a, b). Small nucleolar RNAs are non-coding RNAs consist of 60–300 nt long which accumulate mostly in nucleoli. snoRNAs are involved in various pathophysiological processes. The aberrant expression of snoRNA may induce cell transformation, tumorigenesis, and metastasis. Mt-tRNA plays a role in mitochondrial protein synthesis and oxidative phosphorylation (OXPHOS) to produce energy (ATP) and reactive oxygen species (ROS). Any alteration in the level of Mt-tRNA may have impact on mitochondrial function. The mitochondrial dysfunction has been associated with several lung diseases, such as asbestos-related lung fibrosis and COPD [70, 71]. The Mt-tRNA gene mutations were reported in idiopathic pulmonary fibrosis, lung cancer and hypertension [72–74]. However, we did not perform detailed analysis of Mt-tNRA and snoRNA genes in our samples. In our future studies, we are interested to perform a detailed analysis of Mt-tRNA, snoRNA, snRNA and other differentially expressed small RNA enriched biotypes.

## Conclusions

In conclusion, we have performed a comprehensive plasma exosomes small RNA-sequence analysis that includes exosome isolation, purification, RNA extraction, library preparation, RNA sequencing, and RNA annotation in samples from NS, CS, WP, E-cig users and DS. The isolated RNAs from plasma exosomes were used for small RNA-sequencing analysis. Our data show the enrichment of various non-coding RNAs that include microRNAs, tRNAs, piRNAs, snoRNA, snRNAs, Mt-tRNAs, and other biotypes. Further, the detailed differential expression analysis of microRNAs, tRNAs and piRNA showed significant changes between pairwise comparisons of different groups. Gene set enrichment analysis showed significant changes in the top six enriched functions that were biological pathway, biological process, molecular function, cellular component, site of expression and transcription factor in all groups. The pairwise comparison of tRNAs and piRNA also revealed significant changes in differential expression in all groups. We mapped input reads to human genome and classified to the various small RNA biotypes that showed significant lower counts of Mt-tRNA and snoRNA in all groups. Our comprehensive differential transcriptome analysis done in this study will help to better understand molecular mechanisms of plasma exosome non-coding RNAs, and in developing biomarkers that may be helpful in diagnosis and therapy (theranostics) of lung injury and disease mechanisms in smokers and vapers.

## Supplementary information

**Additional file 1: Supplementary Table 1.** Differential expressed microRNAs from plasma exosomes of E-cigarette users in comparison to non-smokers pairwise comparison.

**Additional file 2: Supplementary Table 2.** Differential expressed microRNAs from plasma exosomes of cigarette smokers in comparison to non-smokers.

**Additional file 3: Supplementary Table 3.** Differential expressed microRNAs from plasma exosomes from waterpipe smokers in comparison to non-smokers.

**Additional file 4: Supplementary Table 4.** Differential expressed microRNAs from plasma exosomes of dual smokers in comparison to non-smokers.

**Additional file 5: Supplementary Table 5.** Differential expressed microRNAs from plasma exosomes of E-cig users in comparison to cigarette smokers.

**Additional file 6: Supplementary Table 6.** Differential expressed microRNAs from plasma exosomes of waterpipe smokers in comparison to cigarette smokers.

**Additional file 7: Supplementary Table 7.** Differential expressed microRNAs from plasma exosomes of dual smokers in comparison to cigarette smokers.

**Additional file 8: Supplementary Table 8.** Differential expressed microRNAs from plasma exosomes of waperpipe smokers in comparison to dual smokers.

**Additional file 9: Supplementary Table 9.** List of target genes of differentially changed microRNA in non-smokers vs. cigarette smokers, non-smokers vs. waterpipe smokers, non-smokers vs. E-cigarette smokers, non-smokers vs. dual smokers, cigarette smokers vs. waterpipe smokers, cigarette smokers vs. E-cigarette smokers, cigarette smokers vs. dual smokers, and dual smokers vs. waterpipe smokers.

**Additional file 10: Supplementary Table 10.** Differential expressed tRNAs from plasma exosomes of cigarette smokers in comparison to non-smokers.

**Additional file 11: Supplementary Table 11.** . Differential expressed tRNAs from plasma exosomes of waterpipe smokers in comparison to non-smokers.

**Additional file 12: Supplementary Table 12.** Differential expressed tRNAs from plasma exosomes of E-cigarette users in comparison to non-smokers.

**Additional file 13: Supplementary Table 13.** Differential expressed tRNAs from plasma exosomes of dual smokers in comparison to non-smokers.

**Additional file 14: Supplementary Table 14.** Differentially expressed piRNAs pairwise comparison between non-smokers vs. cigarette smokers, non-smokers vs. waterpipe smokers, non-smokers vs. E-cig users, non-smokers vs. dual smokers, cigarette smokers vs. waterpipe smokers, cigarette smokers vs. E-cig users, cigarette smokers vs. dual smokers and waterpipe smokers vs. dual smokers.

**Additional file 15: Supplementary Table 15.** Metadata of submitted raw RAN sequencing fastq data for all 36 samples.

**Additional file 16: Supplementary Table 16.** Submission summary of submitted NCBI BioProject database with accession number PRJNA639124.

**Additional file 17: Supplementary Table 17.** URL links of submitted NCBI BioProject database with accession number PRJNA639124.

**Additional file 18: Supplementary Figure 1.** Unedited full immunoblots for Fig. 1A. Suppl. Figure 1 showing full length western blot images as “original western blots”. Full-length gels and blots are shown which are original and unprocessed versions.

**Additional file 19: Supplementary Figure 2**. Volcano plot. Volcano plot showing the relation between *P*-values of the changes in differentially expressed microRNA, and fold change in cigarette smokers, waterpipe smokers, dual smokers and e-cigarette users. (A) Cigarette smokers versus E-cig users. (B) Cigarette smokers versus waterpipe smokers. (C) Cigarette smokers versus dual smokers. (D) Dual smokers versus waterpipe smokers.

**Additional file 20: Supplementary Figure 3.** Hierarchical cluster analysis of differentially expressed miRNAs. (A) Heatmap clustering of the differentially expressed miRNAs significant among cigarette smokers vs. waterpipe smokers. (B) Heatmap clustering of the differentially expressed miRNAs significant among cigarette smokers vs. E-cig users. (C) Heatmap clustering of the differentially expressed miRNAs significant among cigarette smokers vs. dual smokers. (D) Heatmap clustering of the differentially expressed miRNAs significant among waterpipe smokers vs. dual smokers. These top miRNAs were identified based on individual pairwise comparisons (with adjusted *p*-value; *P* < 0.01). The analysis generated using Z scores of the most differentially expressed significant miRNAs. The dendrogram shows clustering of sample groups (cigarette smokers vs. waterpipe smokers, cigarette smokers vs. E-cig users, cigarette smokers vs. dual smokers and waterpipe smokers vs. dual smokers).

**Additional file 21: Supplementary Figure 4.** FunRich gene enrichment analysis for the differentially expressed miRNAs. Here we provide the top 6 enriched: (A) Biological process, (B) Molecular function, (C) Cellular component, (D) Biological pathway, (E) Site of expression, and (F) Transcription factors for the significant miRNAs and possible target genes in cigarette smokers vs. waterpipe smokers, cigarette smokers vs. E-cig users, cigarette smokers vs. dual smokers and dual smokers vs. waterpipe smokers pairwise comparisons.

**Additional file 22: Supplementary Figure 5.** Multidimensional Scaling based on differential tRNA expression in individual samples of non-smokers, cigarette smokers, waterpipe smokers, E-cig users and dual smokers.

**Additional file 23: Supplementary Figure 6.** Volcano plot showing the relation between P-values of the changes in differentially expressed tRNA, and fold change in non-smokers, cigarette, waterpipe, E-cigarette users and dual smokers. (A) Non-smokers versus cigarette smokers. (B) Non-smokers versus waterpipe smokers. (C) Non-smokers versus E-cig users. (D) Non-smokers versus dual smokers.

**Additional file 24: Supplementary Figure 7.** Hierarchical cluster analysis of differentially expressed miRNAs. (A) Heatmap clustering of the differentially expressed tRNAs significant among non-smokers vs. cigarette, smokers. (B) Heatmap clustering of the differentially expressed tRNAs significant among non-smokers vs. waterpipe smokers. (C) Heatmap clustering of the differentially expressed tRNAs significant among non-smokers vs. E-cigarette users. (D) Heatmap clustering of the differentially expressed tRNAs significant among non-smokers vs. dual smokers. These tRNAs were identified based on individual pairwise comparisons (with unadjusted raw *p*-value; *P* < 0.05). The analysis was generated using Z scores of the most differentially expressed significant tRNAs. The dendrogram shows clustering of pairwise comparisons among the different groups (non-smokers vs. cigarette smokers, non-smokers vs. waterpipe smokers, non-smokers vs. E-cigarette users and non-smokers vs. dual smokers).

**Additional file 25: Supplementary Figure 8.** Venn diagram showing the overlap of differentially expressed tRNAs identified from comparing four groups: non-smokers vs. cigarette smokers, non-smokers vs. waterpie smokers, non-smokers vs. E-cig users and non-smokers vs. dual smokers.

**Additional file 26: Supplementary Figure 9.** Multidimensional Scaling based on differential piRNA expression in individual samples of non-smokers, cigarette smokers, waterpipe smokers, E-cig users and dual smokers.

**Additional file 27: Supplementary Figure 10.** Hierarchical cluster analysis of differentially expressed piRNAs. Heatmap clustering of the differentially expressed piRNAs significant among (A) non-smokers vs. cigarette smokers, (B) non-smokers vs. waterpipe smokers, (C) non-smokers vs. E-cigarette users, (D) non-smokers vs. dual smokers (E) cigarette smokers vs. waterpipe smokers, (F) cigarette smokers vs. E-cigarette smokers, (G) cigarette smokers vs. dual smokers, and (H) dual smokers vs. waterpipe smokers. These piRNAs were identified based on individual pairwise comparisons (with unadjusted raw *p*-value; *P* < 0.05). The analysis was generated using Z scores of the most differentially expressed significant piRNAs. The dendrogram shows clustering of pairwise comparisons among the different groups (non-smokers vs. cigarette smokers, non-smokers vs. waterpipe smokers, non-smokers vs. E-cigarette users and non-smokers vs. dual smokers).

**Additional file 28: Supplementary Figure 11.** Venn diagram showing the overlap of differentially expressed piRNAs identified from comparing four groups: non-smokers vs. cigarette smokers, non-smokers vs. waterpipe smokers, non-smokers vs. E-cig users and non-smokers vs. dual smokers.

## Data Availability

All authors confirm the availability of data and materials online/free access to readers as included in this manuscript. All datasets generated and/or analyzed during the current study are included in this manuscript, and are deposited as follow: The raw sequence data in fastq format with associated metadata have been deposited into the NCBI Sequence Read Archive (SRA) public repository. The deposited sequencing data are publically available from the NCBI BioProject database with accession number PRJNA639124. The description of the submitted data are publically available at https://www.ncbi.nlm.nih.gov/bioproject/PRJNA639124. The raw data can be accessed and downloaded from https://www.ncbi.nlm.nih.gov/sra/PRJNA639124. The details of accession numbers and direct web links with the full names of the data banks/repositories corresponding to the hg38 human genome dataset and the human rRNA datasets obtained from web-based sources (based on Norgen Biotek Corp., Canada) used in this study are given below: hg38 Human Genome Sequence Dataset (GTCh38.p5) was publicly available from https://www.gencodegenes.org/human/release_24.html. The rRNA sequences are only listed as 45S, 5S, and mt_rRNA sequences. Based on the data from the OLD EXCERPT, the two rRNA it aligns to are: RNA5S1–201 Ensembl ID: ENSG00000199352 Transcript ID: ENST00000362482.1. The unmapped chromosome assembly: chrUn_GL000220v1 could be found at https://www.ncbi.nlm.nih.gov/nuccore/GL000220.1.

## Abbreviations

E-cigs: E-cigarettes
COPD: Chronic obstructive pulmonary disease
NS: Normal/non-smokers
SM: Cigarette smokers
WP: Waterpipe
DS: Dual smokers
