# Supplementary material for: Exosomal microRNAs are novel circulating biomarkers in cigarette, waterpipe smokers, E-cigarette users and dual smokers
Source: BMC Med Genomics. 2020 Sep 10;13:128. doi: 10.1186/s12920-020-00748-3 (PMC7488025; doi:10.1186/s12920-020-00748-3)
Supplement: Supplementary file 1 — Additional file 1: Supplementary Table 1. Differential expressed microRNAs from plasma exosomes of E-cigarette users in comparison to non-smokers pairwise comparison. [file 12920_2020_748_MOESM1_ESM.docx]

**Supplementary Table 1. Differential expressed microRNAs from plasma exosomes of non-smokers in comparison to E-Cig users**

| **MicroRNA** | **log2 Fold Change** | **t-test p-value** | **FDR adjusted p-value** |
| --- | --- | --- | --- |
| hsa-miR-365a-3p\|hsa-miR-365b-3p | 24.31696667 | 2.49E-34 | 1.18E-31 |
| hsa-miR-362-5p | -44.56316991 | 9.82E-23 | 2.32E-20 |
| hsa-miR-29b-3p | -24.33979833 | 2.75E-17 | 4.33E-15 |
| hsa-let-7f-5p | 1.411521404 | 9.74E-08 | 1.15E-05 |
| hsa-miR-1299 | 20.10058816 | 1.50E-07 | 1.42E-05 |
| hsa-miR-21-5p | 1.302931807 | 7.13E-07 | 5.29E-05 |
| hsa-let-7i-5p | 1.367958903 | 7.84E-07 | 5.29E-05 |
| hsa-let-7a-5p | 1.534736775 | 1.52E-06 | 8.96E-05 |
| hsa-miR-30a-5p | 1.502742916 | 1.56E-05 | 0.000735 |
| hsa-miR-193b-3p | 8.77133019 | 1.41E-05 | 0.000735 |
| hsa-miR-100-5p | 1.252907903 | 8.37E-05 | 0.003478 |
| hsa-miR-423-3p | 1.482078655 | 8.84E-05 | 0.003478 |
| hsa-miR-30c-5p | 1.499774738 | 0.000276283 | 0.010031 |
| hsa-miR-451a | -1.79956883 | 0.000781282 | 0.02634 |
| hsa-miR-143-3p | 1.038901917 | 0.00091283 | 0.028724 |
| hsa-miR-224-5p | 2.01472867 | 0.001192386 | 0.035175 |
| hsa-miR-30e-5p | -1.300417432 | 0.001717024 | 0.047673 |

Upregulated: 13, Downregulated: 4
