# Supplementary material for: Exosomal microRNAs are novel circulating biomarkers in cigarette, waterpipe smokers, E-cigarette users and dual smokers
Source: BMC Med Genomics. 2020 Sep 10;13:128. doi: 10.1186/s12920-020-00748-3 (PMC7488025; doi:10.1186/s12920-020-00748-3)
Supplement: Supplementary file 2 — Additional file 2: Supplementary Table 2. Differential expressed microRNAs from plasma exosomes of cigarette smokers in comparison to non-smokers. [file 12920_2020_748_MOESM2_ESM.docx]

**Supplementary Table 2. Differential expressed microRNAs from plasma exosomes of non-smokers in comparison to cigarette smokers**

| **MicroRNA** | **log2 Fold change** | **t-test p-value** | **FDR adjusted p-value** |
| --- | --- | --- | --- |
| hsa-miR-29b-3p | -23.576 | 2.61E-16 | 1.23E-13 |
| hsa-miR-532-5p | 19.79841 | 1.94E-08 | 4.57E-06 |
| hsa-let-7i-5p | 1.450293 | 1.63E-07 | 1.92E-05 |
| hsa-miR-2355-5p | 19.65169 | 1.53E-07 | 1.92E-05 |
| hsa-let-7f-5p | 1.247658 | 2.43E-06 | 0.000191 |
| hsa-let-7a-5p | 1.504581 | 2.42E-06 | 0.000191 |
| hsa-miR-21-5p | 1.20414 | 4.59E-06 | 0.00031 |
| hsa-miR-149-5p | 20.29544 | 8.09E-06 | 0.000478 |
| hsa-miR-30a-5p | 1.533732 | 1.03E-05 | 0.000542 |
| hsa-miR-143-3p | 1.371514 | 1.20E-05 | 0.000565 |
| hsa-miR-144-5p | 15.35903 | 1.85E-05 | 0.000793 |
| hsa-let-7g-5p | 1.150642 | 8.29E-05 | 0.003261 |
| hsa-miR-10b-5p | -1.75655 | 9.15E-05 | 0.003322 |
| hsa-miR-146b-5p | -1.47302 | 0.000201 | 0.006766 |
| hsa-miR-23a-3p | 0.860353 | 0.000224 | 0.007057 |
| hsa-miR-100-5p | 1.15055 | 0.000303 | 0.00843 |
| hsa-miR-30c-5p | 1.487793 | 0.000304 | 0.00843 |
| hsa-miR-29a-3p | -2.58574 | 0.000391 | 0.010263 |
| hsa-miR-92a-3p | 0.796846 | 0.00057 | 0.014156 |
| hsa-miR-320b | -2.13025 | 0.000608 | 0.014349 |
| hsa-miR-125b-5p | 1.305183 | 0.00102 | 0.022915 |
| hsa-miR-126-3p | -1.38763 | 0.001223 | 0.026229 |
| hsa-miR-144-3p | -1.72478 | 0.001767 | 0.03627 |
| hsa-miR-186-5p | -0.71614 | 0.002206 | 0.043391 |

Upregulated: 16, Downregulated: 8.
