# Supplementary material for: Exosomal microRNAs are novel circulating biomarkers in cigarette, waterpipe smokers, E-cigarette users and dual smokers
Source: BMC Med Genomics. 2020 Sep 10;13:128. doi: 10.1186/s12920-020-00748-3 (PMC7488025; doi:10.1186/s12920-020-00748-3)
Supplement: Supplementary file 3 — Additional file 3: Supplementary Table 3. Differential expressed microRNAs from plasma exosomes from waterpipe smokers in comparison to non-smokers. [file 12920_2020_748_MOESM3_ESM.docx]

**Supplementary Table 3. Differential expressed microRNAs from plasma exosomes from non-smokers in comparison to waterpipe smokers**

| **MicroRNA** | **log2 Fold Change** | **t-test p-value** | **FDR adjusted p-value** |
| --- | --- | --- | --- |
| hsa-miR-2355-5p | 39.81895 | 2.05E-26 | 9.67E-24 |
| hsa-miR-362-5p | -45.3582 | 1.67E-23 | 3.95E-21 |
| hsa-miR-29b-3p | -24.6204 | 1.19E-17 | 1.87E-15 |
| hsa-miR-582-5p | 23.15152 | 7.50E-11 | 8.85E-09 |
| hsa-miR-149-5p | 29.22476 | 1.31E-10 | 1.24E-08 |
| hsa-miR-1299 | 19.61721 | 2.96E-07 | 2.33E-05 |
| hsa-miR-1-3p | 7.566971 | 7.31E-07 | 4.93E-05 |
| hsa-let-7i-5p | 1.085857 | 8.84E-05 | 0.005213 |
| hsa-miR-320b | -2.29686 | 0.000226 | 0.01184 |
| hsa-miR-21-5p | 0.933212 | 0.000384 | 0.015101 |
| hsa-let-7f-5p | 0.941049 | 0.000378 | 0.015101 |
| hsa-miR-143-3p | 1.124826 | 0.000332 | 0.015101 |
| hsa-miR-320d | -4.57674 | 0.000509 | 0.018471 |
| hsa-let-7a-5p | 1.020428 | 0.001386 | 0.045733 |
| hsa-miR-30a-5p | 1.100946 | 0.00155 | 0.045733 |
| hsa-let-7g-5p | 0.925974 | 0.00155 | 0.045733 |

Upregulated: 12, Downregulated: 4.
