# Supplementary material for: Exosomal microRNAs are novel circulating biomarkers in cigarette, waterpipe smokers, E-cigarette users and dual smokers
Source: BMC Med Genomics. 2020 Sep 10;13:128. doi: 10.1186/s12920-020-00748-3 (PMC7488025; doi:10.1186/s12920-020-00748-3)
Supplement: Supplementary file 4 — Additional file 4: Supplementary Table 4. Differential expressed microRNAs from plasma exosomes of dual smokers in comparison to non-smokers. [file 12920_2020_748_MOESM4_ESM.docx]

**Supplementary Table 4. Differential expressed microRNAs from plasma exosomes of non-smokers in comparison to ual smokers**

| **MicroRNA** | **log2 Fold Change** | **t-test p-value** | **FDR adjusted p-value** |
| --- | --- | --- | --- |
| hsa-miR-362-5p | -44.1156 | 2.76E-22 | 1.30E-19 |
| hsa-miR-29b-3p | -21.546 | 8.99E-14 | 2.12E-11 |
| hsa-miR-149-5p | 29.29248 | 1.19E-10 | 1.87E-08 |
| hsa-let-7i-5p | 1.35993 | 9.16E-07 | 0.000108 |
| hsa-miR-21-5p | 1.138827 | 1.47E-05 | 0.00139 |
| hsa-miR-144-3p | -2.36741 | 1.78E-05 | 0.001397 |
| hsa-miR-143-3p | 1.284561 | 4.17E-05 | 0.0024 |
| hsa-miR-30a-5p | 1.418061 | 4.58E-05 | 0.0024 |
| hsa-miR-30c-5p | 1.693464 | 4.23E-05 | 0.0024 |
| hsa-let-7f-5p | 1.052664 | 7.02E-05 | 0.003312 |
| hsa-miR-451a | -2.06707 | 0.000114 | 0.004893 |
| hsa-miR-10b-5p | -1.66326 | 0.000212 | 0.0077 |
| hsa-let-7a-5p | 1.182338 | 0.000212 | 0.0077 |
| hsa-miR-424-3p | 16.01973 | 0.000284 | 0.009574 |
| hsa-miR-139-5p | 16.41286 | 0.000349 | 0.010992 |
| hsa-miR-100-5p | 1.094238 | 0.000592 | 0.017454 |
| hsa-miR-25-3p | -0.63808 | 0.000783 | 0.021753 |
| hsa-let-7g-5p | 0.959653 | 0.00104 | 0.027277 |
| hsa-miR-23a-3p | 0.724237 | 0.00192 | 0.047694 |
| hsa-miR-192-5p | -1.21216 | 0.0021 | 0.049555 |

Upregulated:13, Downregulated: 7.
