# Supplementary material for: Exosomal microRNAs are novel circulating biomarkers in cigarette, waterpipe smokers, E-cigarette users and dual smokers
Source: BMC Med Genomics. 2020 Sep 10;13:128. doi: 10.1186/s12920-020-00748-3 (PMC7488025; doi:10.1186/s12920-020-00748-3)
Supplement: Supplementary file 5 — Additional file 5: Supplementary Table 5. Differential expressed microRNAs from plasma exosomes of E-cig users in comparison to cigarette smokers. [file 12920_2020_748_MOESM5_ESM.docx]

**Supplementary Table 5. Differential expressed microRNAs from plasma exosomes of E-cig users in comparison to cigarette smokers**

| **MicroRNA** | **log2 Fold Change** | **t-test p-value** | **FDR adjusted p-values** |
| --- | --- | --- | --- |
| hsa-miR-365a-3p | -24.1251 | 4.36E-32 | 2.06E-29 |
| hsa-miR-1299 | -24.0144 | 1.11E-09 | 2.63E-07 |
| hsa-miR-532-5p | 19.41787 | 9.37E-08 | 1.47E-05 |
| hsa-miR-30e-5p | 2.239339 | 1.77E-07 | 2.08E-05 |
| hsa-miR-2355-5p | 19.62366 | 3.79E-07 | 3.58E-05 |
| hsa-miR-362-5p | 19.67676 | 2.76E-05 | 0.002173 |
| hsa-miR-193b-3p | -7.85837 | 0.000155 | 0.01045 |
| hsa-miR-186-5p | -0.89544 | 0.000215 | 0.011296 |
| hsa-miR-144-5p | 13.84101 | 0.00021 | 0.011296 |

Upregulated: 5, Downregulated: 4.
