# Supplementary material for: Exosomal microRNAs are novel circulating biomarkers in cigarette, waterpipe smokers, E-cigarette users and dual smokers
Source: BMC Med Genomics. 2020 Sep 10;13:128. doi: 10.1186/s12920-020-00748-3 (PMC7488025; doi:10.1186/s12920-020-00748-3)
Supplement: Supplementary file 6 — Additional file 6: Supplementary Table 6. Differential expressed microRNAs from plasma exosomes of waterpipe smokers in comparison to cigarette smokers. [file 12920_2020_748_MOESM6_ESM.docx]

**Supplementary Table 6. Differential expressed microRNAs from plasma exosomes of waterpipe smokers in comparison to cigarette smokers**

| **MicroRNA** | **log2 Fold Change** | **t-test p-value** | **FDR adjusted p-value** |
| --- | --- | --- | --- |
| hsa-miR-582-5p | -22.6318 | 6.81E-10 | 3.21E-07 |
| hsa-miR-1299 | -23.531 | 2.38E-09 | 5.62E-07 |
| hsa-miR-532-5p | 21.30146 | 4.58E-09 | 7.20E-07 |
| hsa-miR-144-5p | 19.42889 | 1.11E-07 | 1.31E-05 |
| hsa-miR-362-5p | 20.47183 | 1.29E-05 | 0.001215 |
| hsa-miR-1-3p | -6.72446 | 1.78E-05 | 0.001401 |

Upregulated: 3, Downregulated: 3.
