# Supplementary material for: Exosomal microRNAs are novel circulating biomarkers in cigarette, waterpipe smokers, E-cigarette users and dual smokers
Source: BMC Med Genomics. 2020 Sep 10;13:128. doi: 10.1186/s12920-020-00748-3 (PMC7488025; doi:10.1186/s12920-020-00748-3)
Supplement: Supplementary file 7 — Additional file 7: Supplementary Table 7. Differential expressed microRNAs from plasma exosomes of dual smokers in comparison to cigarette smokers. [file 12920_2020_748_MOESM7_ESM.docx]

**Supplementary Table 7. Differential expressed microRNAs from plasma exosomes of cigarette smokers in comparison to dual smokers**

| **MicroRNA** | **log2 Fold Change** | **t-test p-value** | **FDR adjusted p-value** |
| --- | --- | --- | --- |
| hsa-miR-144-5p | -21.4076 | 4.74E-09 | 2.24E-06 |
| hsa-miR-532-5p | -20.7478 | 1.14E-08 | 2.70E-06 |
| hsa-miR-2355-5p | -19.263 | 6.30E-07 | 9.92E-05 |
| hsa-miR-424-3p | 20.26167 | 7.10E-06 | 0.000837 |
| hsa-miR-362-5p | -19.2292 | 4.23E-05 | 0.003995 |

Upregulated: 1, Downregulated: 4.
