# Supplementary material for: Exosomal microRNAs are novel circulating biomarkers in cigarette, waterpipe smokers, E-cigarette users and dual smokers
Source: BMC Med Genomics. 2020 Sep 10;13:128. doi: 10.1186/s12920-020-00748-3 (PMC7488025; doi:10.1186/s12920-020-00748-3)
Supplement: Supplementary file 8 — Additional file 8: Supplementary Table 8. Differential expressed microRNAs from plasma exosomes of waperpipe smokers in comparison to dual smokers. [file 12920_2020_748_MOESM8_ESM.docx]

**Supplementary Table 8. Differential expressed microRNAs from plasma exosomes of waperpipe smokers in comparison to dual smokers**

| **MicroRNA** | **log2 Fold Change** | **t-test p-value** | **FDR adjusted p-value** |
| --- | --- | --- | --- |
| hsa-miR-2355-5p | -39.4303 | 2.05E-24 | 9.66E-22 |
| hsa-miR-582-5p | -21.2517 | 7.13E-09 | 1.68E-06 |
| hsa-miR-1299 | -21.2967 | 6.76E-08 | 1.06E-05 |
| hsa-miR-424-3p | 21.71333 | 1.48E-06 | 0.000174 |
| hsa-miR-139-5p | 22.07554 | 2.56E-06 | 0.000242 |
| hsa-miR-1-3p | -7.29498 | 3.27E-06 | 0.000257 |

Upregulated: 2, Downregulated: 4.
