# Supplementary material for: Exosomal microRNAs are novel circulating biomarkers in cigarette, waterpipe smokers, E-cigarette users and dual smokers
Source: BMC Med Genomics. 2020 Sep 10;13:128. doi: 10.1186/s12920-020-00748-3 (PMC7488025; doi:10.1186/s12920-020-00748-3)
Supplement: Supplementary file 10 — Additional file 10: Supplementary Table 10. Differential expressed tRNAs from plasma exosomes of cigarette smokers in comparison to non-smokers. [file 12920_2020_748_MOESM10_ESM.docx]

Supplementary Table 10. Differential expressed tRNAs from plasma exosomes of non-smokers in comparison to cigarette smokers

| tRNA | Log2 fold change | P value | Adjusted p value |
| --- | --- | --- | --- |
| Val | 2.3406 | 1.52E-10 | 3.65E-09 |
| Glu | 2.0124 | 6.35E-010 | 7.62E-09 |
| Asp | 3.031 | 4.61E-08 | 3.69E-07 |
| Gly | 1.930 | 9.17E-07 | 5.50E-07 |
| Arg | 2.7496 | 0.000238 | 0.001359 |
| Cys | -0.8091 | 0.000502 | 0.002009 |
| His | 1.6837 | 0.008763 | 0.030044 |
