# Supplementary material for: Exosomal microRNAs are novel circulating biomarkers in cigarette, waterpipe smokers, E-cigarette users and dual smokers
Source: BMC Med Genomics. 2020 Sep 10;13:128. doi: 10.1186/s12920-020-00748-3 (PMC7488025; doi:10.1186/s12920-020-00748-3)
Supplement: Supplementary file 11 — Additional file 11: Supplementary Table 11. . Differential expressed tRNAs from plasma exosomes of waterpipe smokers in comparison to non-smokers. [file 12920_2020_748_MOESM11_ESM.docx]

Supplementary Table 11. Differential expressed tRNAs from plasma exosomes of non-smokers in comparison to waterpipe smokers

| tRNA | Log2 fold change | P value | Adjusted p value |
| --- | --- | --- | --- |
| Val | 2.2759 | 4.94E-10 | 1.19E-08 |
| Glu | 1.9474 | 2.23E-09 | 2.67E-08 |
| Gly | 1.7440 | 1.39E-06 | 1.12E-05 |
| Asp | 2.1636 | 9.08E-05 | 0.000545 |
| His | 1.9728 | 0.002153 | 0.009053 |
| Arg | 2.3120 | 0.002263 | 0.009053 |
| Cys | -0.6325 | 0.006589 | 0.022591 |
