# Supplementary material for: Exosomal microRNAs are novel circulating biomarkers in cigarette, waterpipe smokers, E-cigarette users and dual smokers
Source: BMC Med Genomics. 2020 Sep 10;13:128. doi: 10.1186/s12920-020-00748-3 (PMC7488025; doi:10.1186/s12920-020-00748-3)
Supplement: Supplementary file 12 — Additional file 12: Supplementary Table 12. Differential expressed tRNAs from plasma exosomes of E-cigarette users in comparison to non-smokers. [file 12920_2020_748_MOESM12_ESM.docx]

Supplementary Table 12. Differential expressed tRNAs from plasma exosomes of non-smokers in comparison to E-cigarette users

| tRNA | Log2 fold change | P value | Adjusted p value |
| --- | --- | --- | --- |
| Val | 2.4230 | 3.47E-11 | 8.33E-10 |
| Glu | 1.8154 | 2.44E-08 | 2.93E-07 |
| Gly | 1.6895 | 2.94E-06 | 2.35E-05 |
| Arg | 2.3848 | 0.00163 | 0.009778 |
| His | 1.8243 | 0.004523 | 0.02171 |
| Asp | 1.4683 | 0.007691 | 0.030764 |
| Cys | -0.5851 | 0.011633 | 0.039884 |
