# Supplementary material for: Exosomal microRNAs are novel circulating biomarkers in cigarette, waterpipe smokers, E-cigarette users and dual smokers
Source: BMC Med Genomics. 2020 Sep 10;13:128. doi: 10.1186/s12920-020-00748-3 (PMC7488025; doi:10.1186/s12920-020-00748-3)
Supplement: Supplementary file 13 — Additional file 13: Supplementary Table 13. Differential expressed tRNAs from plasma exosomes of dual smokers in comparison to non-smokers. [file 12920_2020_748_MOESM13_ESM.docx]

Supplementary Table 13. Differential expressed tRNAs from plasma exosomes of non-smokers in comparison to dual smokers

| tRNA | Log2 fold change | P vaule | Adjusted p value |
| --- | --- | --- | --- |
| Glu | 2.180035 | 2.19E-11 | 5.26E-10 |
| Val | 2.095814 | 9.91E-09 | 1.19E-07 |
| Gly | 1.670659 | 3.78E-06 | 3.03E-05 |
| Asp | 2.206589 | 6.58E-05 | 0.000395 |
| His | 1.968093 | 0.002207 | 0.10594 |
| Ile | 2.975488 | 0.007315 | 0.029258 |
| Arg | 1.946265 | 0.010103 | 0.034638 |
| Cys | -0.56381 | 0.015473 | 0.04642 |
