# Supplementary material for: Exosomal microRNAs are novel circulating biomarkers in cigarette, waterpipe smokers, E-cigarette users and dual smokers
Source: BMC Med Genomics. 2020 Sep 10;13:128. doi: 10.1186/s12920-020-00748-3 (PMC7488025; doi:10.1186/s12920-020-00748-3)
Supplement: Supplementary file 18 — Additional file 18: Supplementary Figure 1. Unedited full immunoblots for Fig. 1A. Suppl. Figure 1 showing full length western blot images as “original western blots”. Full-length gels and blots are shown which are original and unprocessed versions. [file 12920_2020_748_MOESM18_ESM.pptx]

## Slide 1
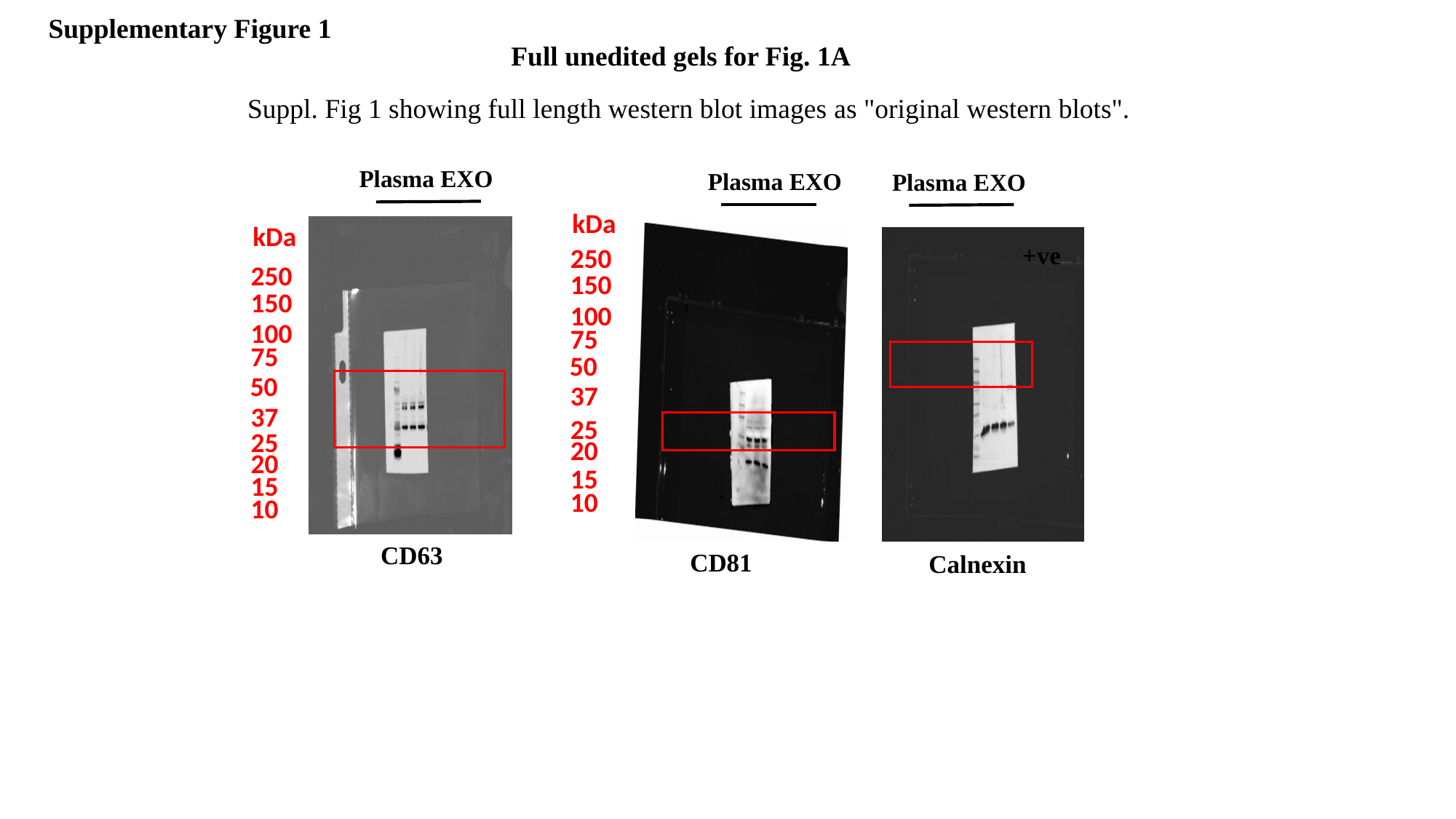

Supplementary Figure 1
Full unedited gels for Fig. 1A
Suppl. Fig 1 showing full length western blot images as "original western blots".
Plasma EXO
Plasma EXO
Plasma EXO
kDa
kDa
+ve
250
250
150
150
100
100
75
75
50
50
37
37
25
25
20
20
15
15
10
10
CD63
CD81
Calnexin
