# Supplementary material for: Exosomal microRNAs are novel circulating biomarkers in cigarette, waterpipe smokers, E-cigarette users and dual smokers
Source: BMC Med Genomics. 2020 Sep 10;13:128. doi: 10.1186/s12920-020-00748-3 (PMC7488025; doi:10.1186/s12920-020-00748-3)
Supplement: Supplementary file 19 — Additional file 19: Supplementary Figure 2. Volcano plot. Volcano plot showing the relation between P-values of the changes in differentially expressed microRNA, and fold change in cigarette smokers, waterpipe smokers, dual smokers and e-cigarette users. (A) Cigarette smokers versus E-cig users. (B) Cigarette smokers versus waterpipe smokers. (C) Cigarette smokers versus dual smokers. (D) Dual smokers versus waterpipe smokers. [file 12920_2020_748_MOESM19_ESM.pptx]

## Slide 1
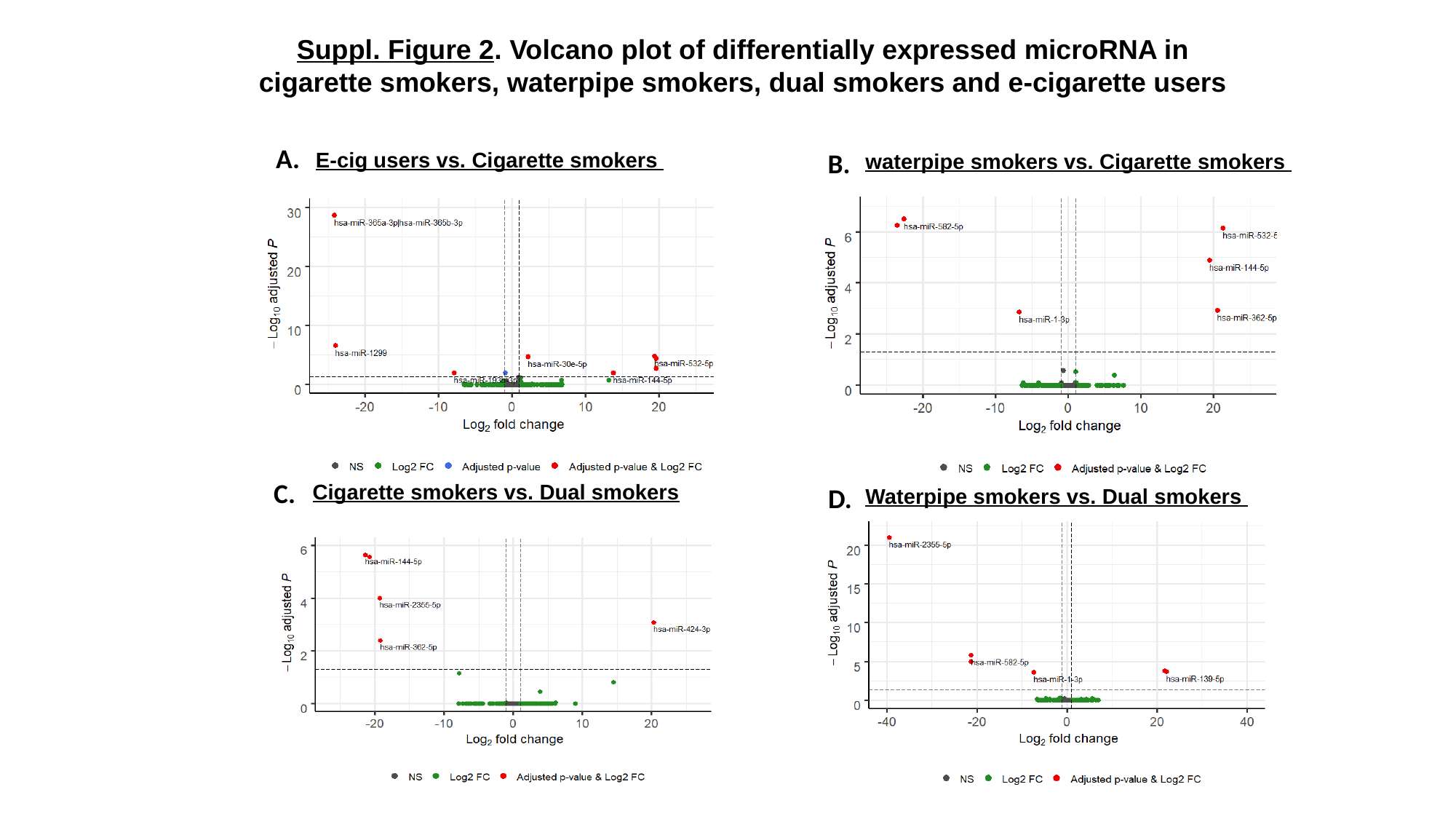

Suppl. Figure 2. Volcano plot of differentially expressed microRNA in cigarette smokers, waterpipe smokers, dual smokers and e-cigarette users
A.
E-cig users vs. Cigarette smokers
B.
waterpipe smokers vs. Cigarette smokers
C.
Cigarette smokers vs. Dual smokers
D.
Waterpipe smokers vs. Dual smokers
