# Supplementary material for: Exosomal microRNAs are novel circulating biomarkers in cigarette, waterpipe smokers, E-cigarette users and dual smokers
Source: BMC Med Genomics. 2020 Sep 10;13:128. doi: 10.1186/s12920-020-00748-3 (PMC7488025; doi:10.1186/s12920-020-00748-3)
Supplement: Supplementary file 20 — Additional file 20: Supplementary Figure 3. Hierarchical cluster analysis of differentially expressed miRNAs. (A) Heatmap clustering of the differentially expressed miRNAs significant among cigarette smokers vs. waterpipe smokers. (B) Heatmap clustering of the differentially expressed miRNAs significant among cigarette smokers vs. E-cig users. (C) Heatmap clustering of the differentially expressed miRNAs significant among cigarette smokers vs. dual smokers. (D) Heatmap clustering of the differentially expressed miRNAs significant among waterpipe smokers vs. dual smokers. These top miRNAs were identified based on individual pairwise comparisons (with adjusted p-value; P < 0.01). The analysis generated using Z scores of the most differentially expressed significant miRNAs. The dendrogram shows clustering of sample groups (cigarette smokers vs. waterpipe smokers, cigarette smokers vs. E-cig users, cigarette smokers vs. dual smokers and waterpipe smokers vs. dual smokers). [file 12920_2020_748_MOESM20_ESM.pptx]

## Slide 1
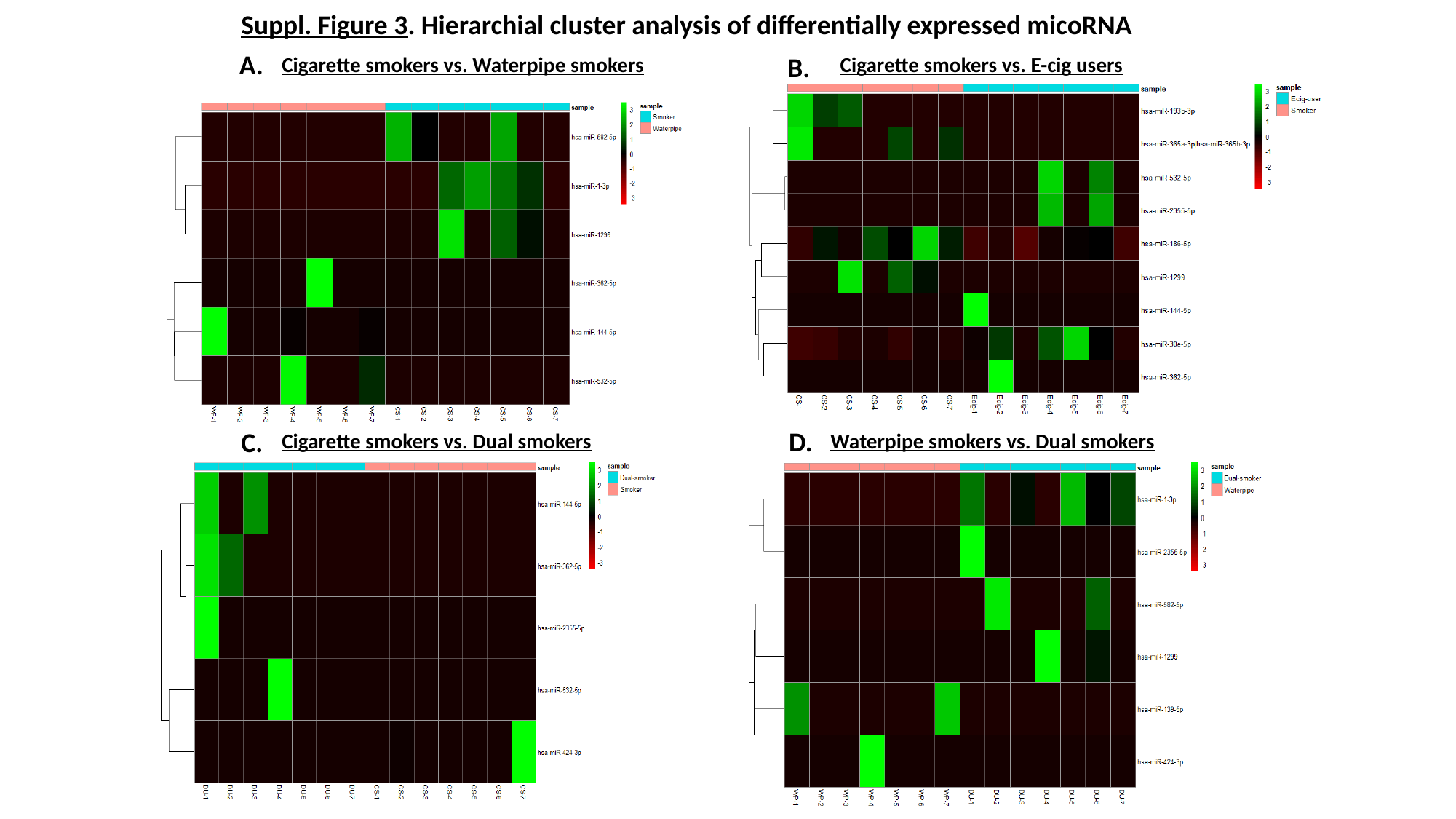

Suppl. Figure 3. Hierarchial cluster analysis of differentially expressed micoRNA
A.
B.
Cigarette smokers vs. E-cig users
Cigarette smokers vs. Waterpipe smokers
D.
C.
Cigarette smokers vs. Dual smokers
Waterpipe smokers vs. Dual smokers
