# Supplementary material for: Exosomal microRNAs are novel circulating biomarkers in cigarette, waterpipe smokers, E-cigarette users and dual smokers
Source: BMC Med Genomics. 2020 Sep 10;13:128. doi: 10.1186/s12920-020-00748-3 (PMC7488025; doi:10.1186/s12920-020-00748-3)
Supplement: Supplementary file 21 — Additional file 21: Supplementary Figure 4. FunRich gene enrichment analysis for the differentially expressed miRNAs. Here we provide the top 6 enriched: (A) Biological process, (B) Molecular function, (C) Cellular component, (D) Biological pathway, (E) Site of expression, and (F) Transcription factors for the significant miRNAs and possible target genes in cigarette smokers vs. waterpipe smokers, cigarette smokers vs. E-cig users, cigarette smokers vs. dual smokers and dual smokers vs. waterpipe smokers pairwise comparisons. [file 12920_2020_748_MOESM21_ESM.pptx]

## Slide 1
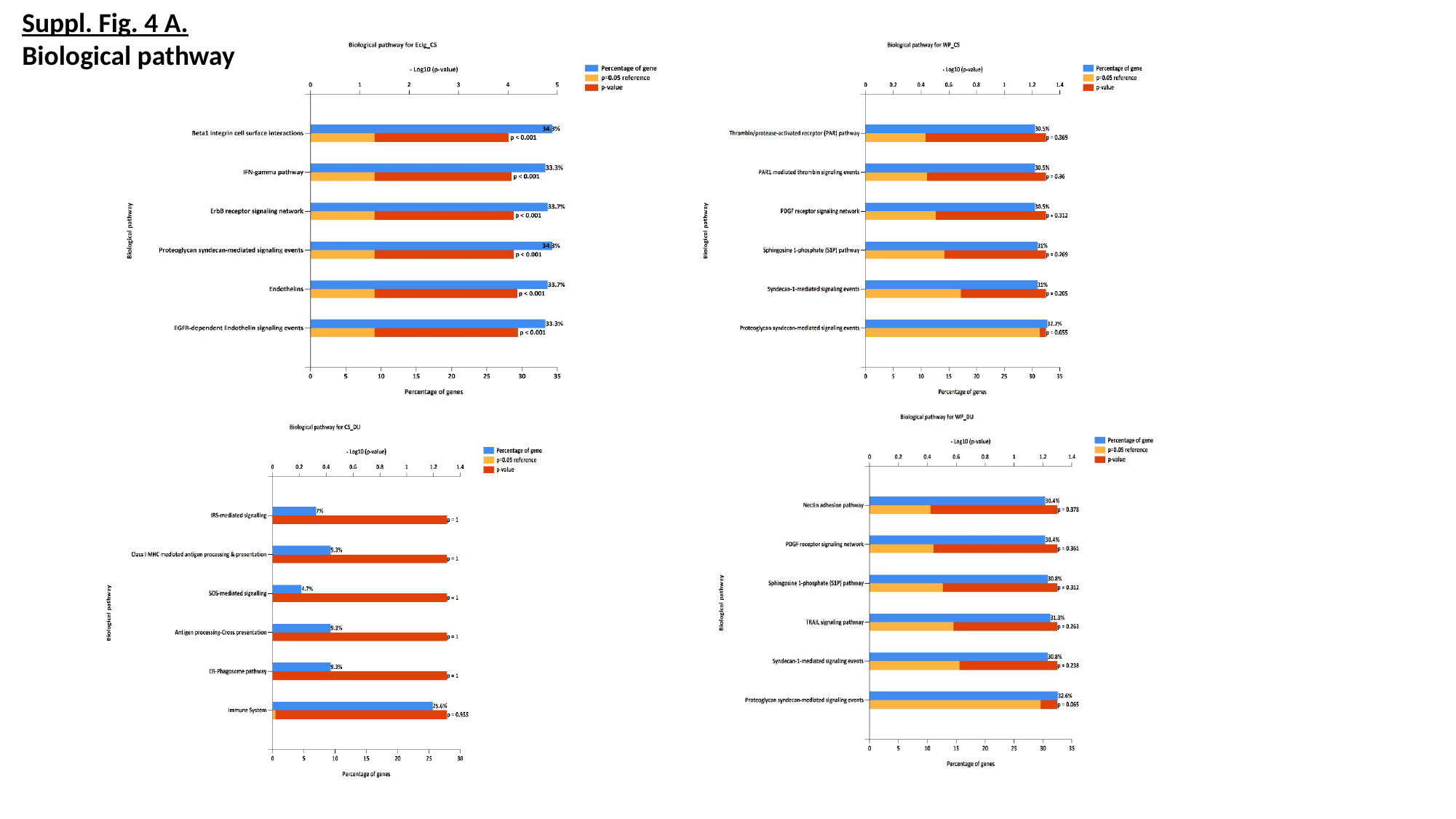

Suppl. Fig. 4 A.
Biological pathway

## Slide 2
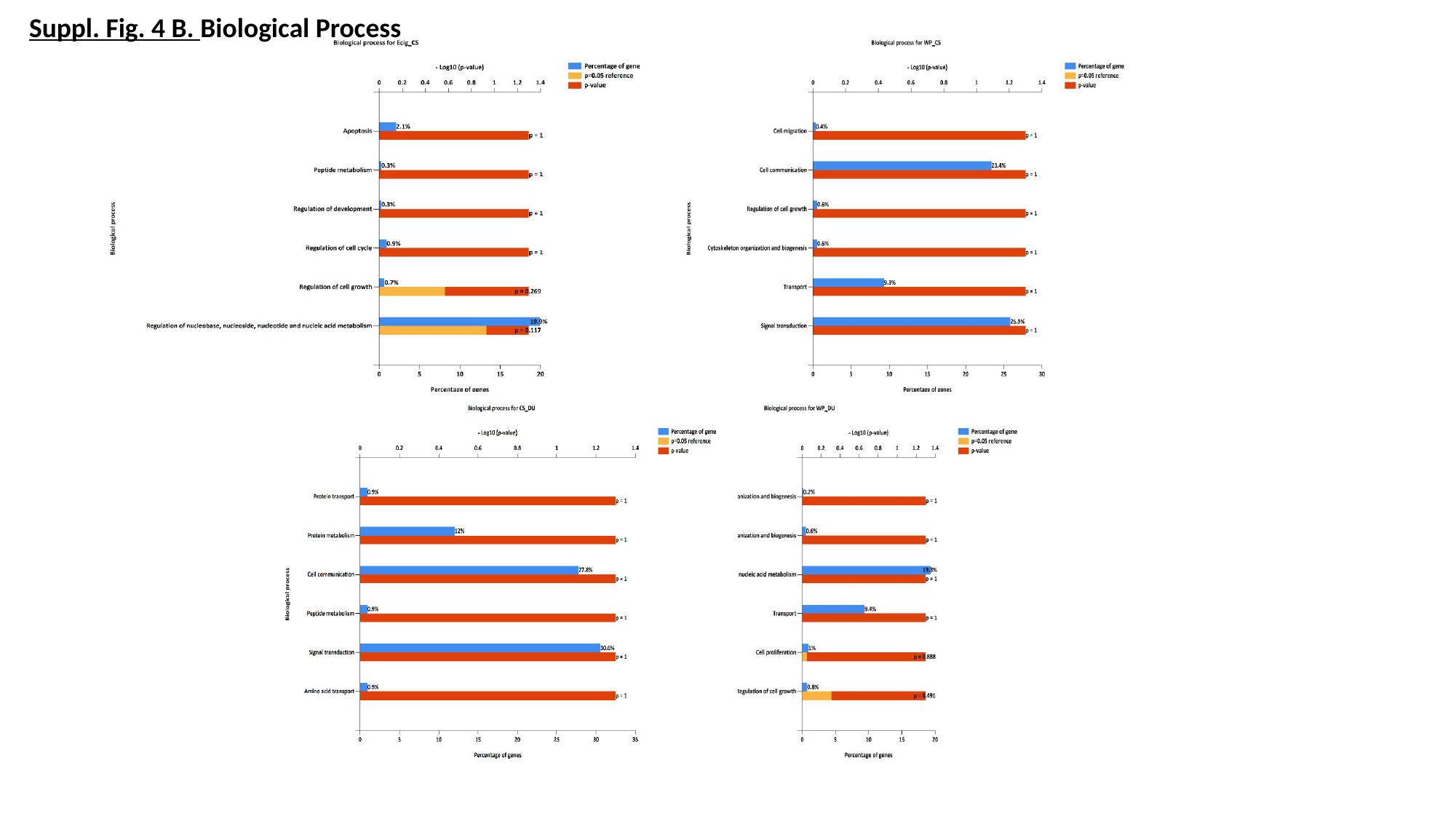

Suppl. Fig. 4 B. Biological Process

## Slide 3
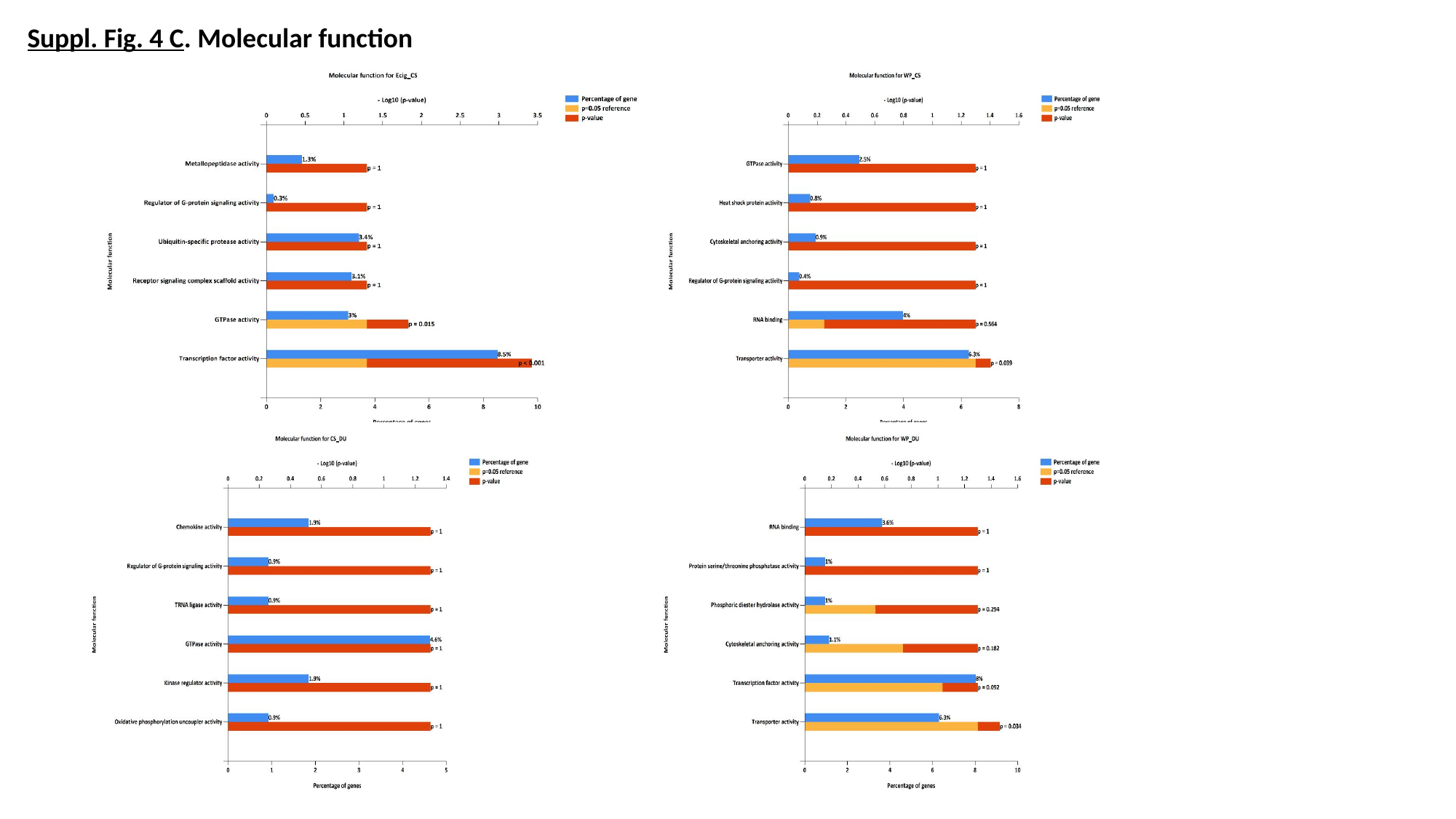

Suppl. Fig. 4 C. Molecular function

## Slide 4
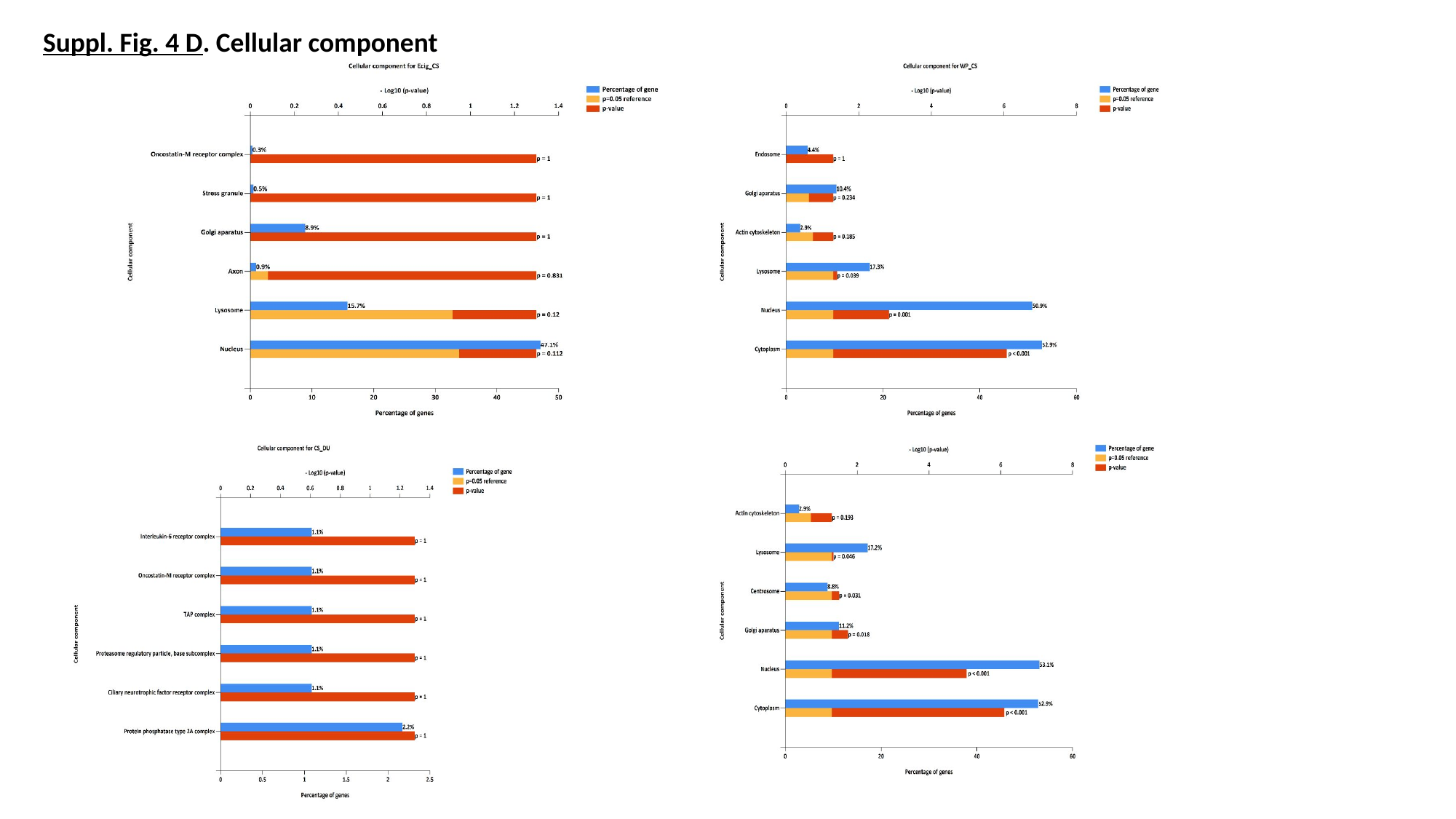

Suppl. Fig. 4 D. Cellular component

## Slide 5
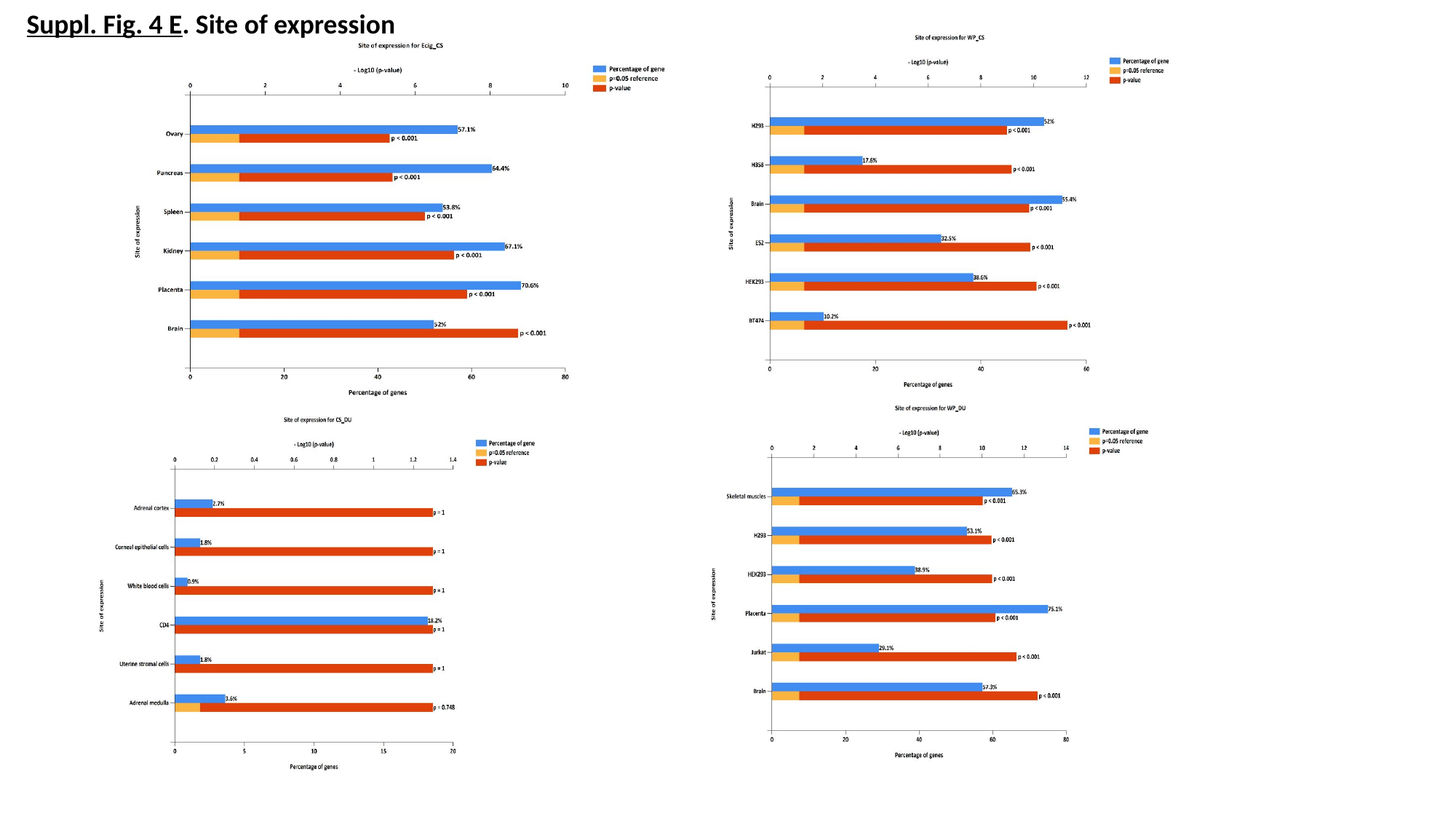

Suppl. Fig. 4 E. Site of expression

## Slide 6
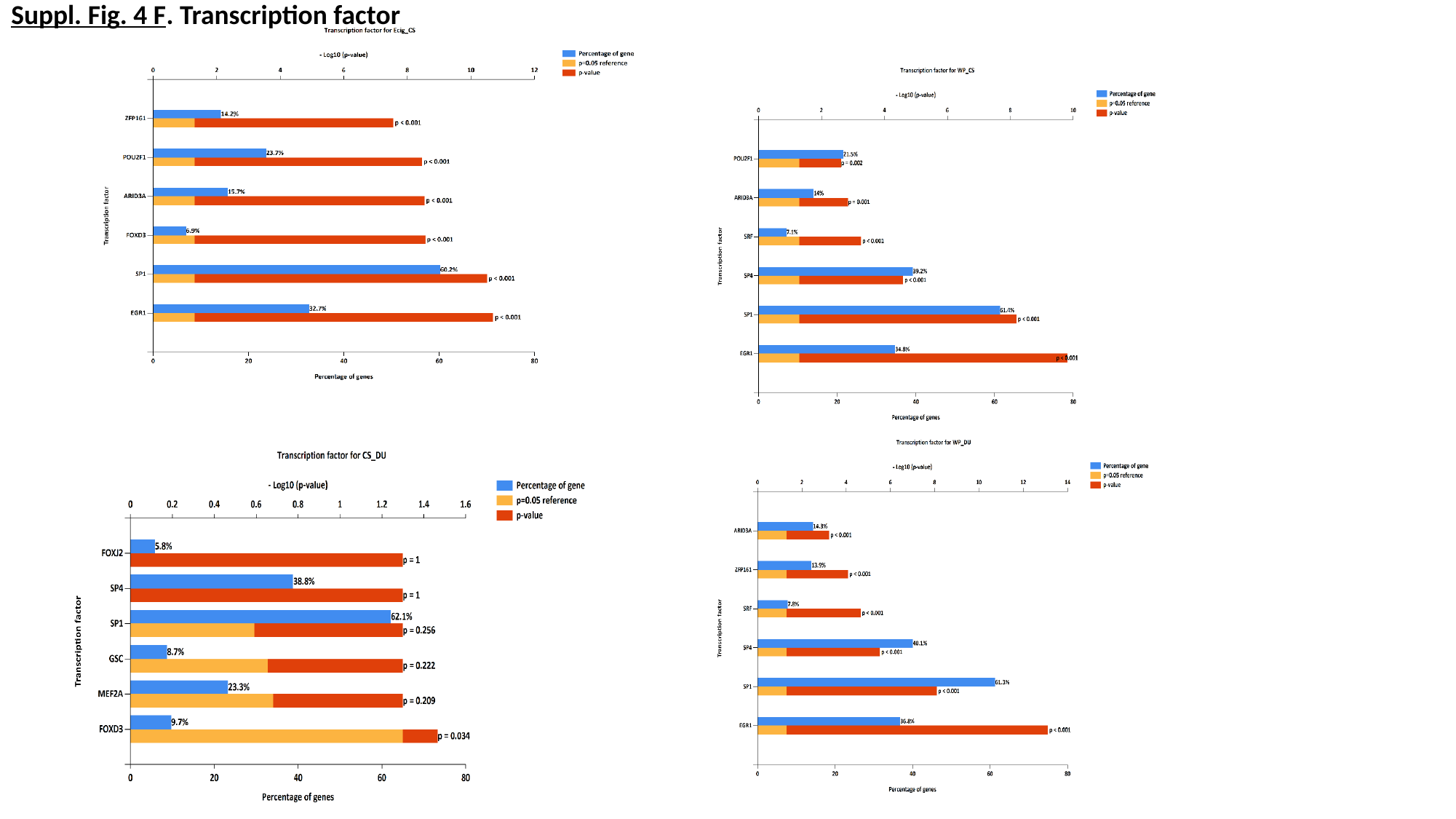

Suppl. Fig. 4 F. Transcription factor
