# Supplementary figures and images for: Exosomal microRNAs are novel circulating biomarkers in cigarette, waterpipe smokers, E-cigarette users and dual smokers
Source: BMC Med Genomics. 2020 Sep 10;13:128. doi: 10.1186/s12920-020-00748-3 (PMC7488025; doi:10.1186/s12920-020-00748-3)

## Slide 1
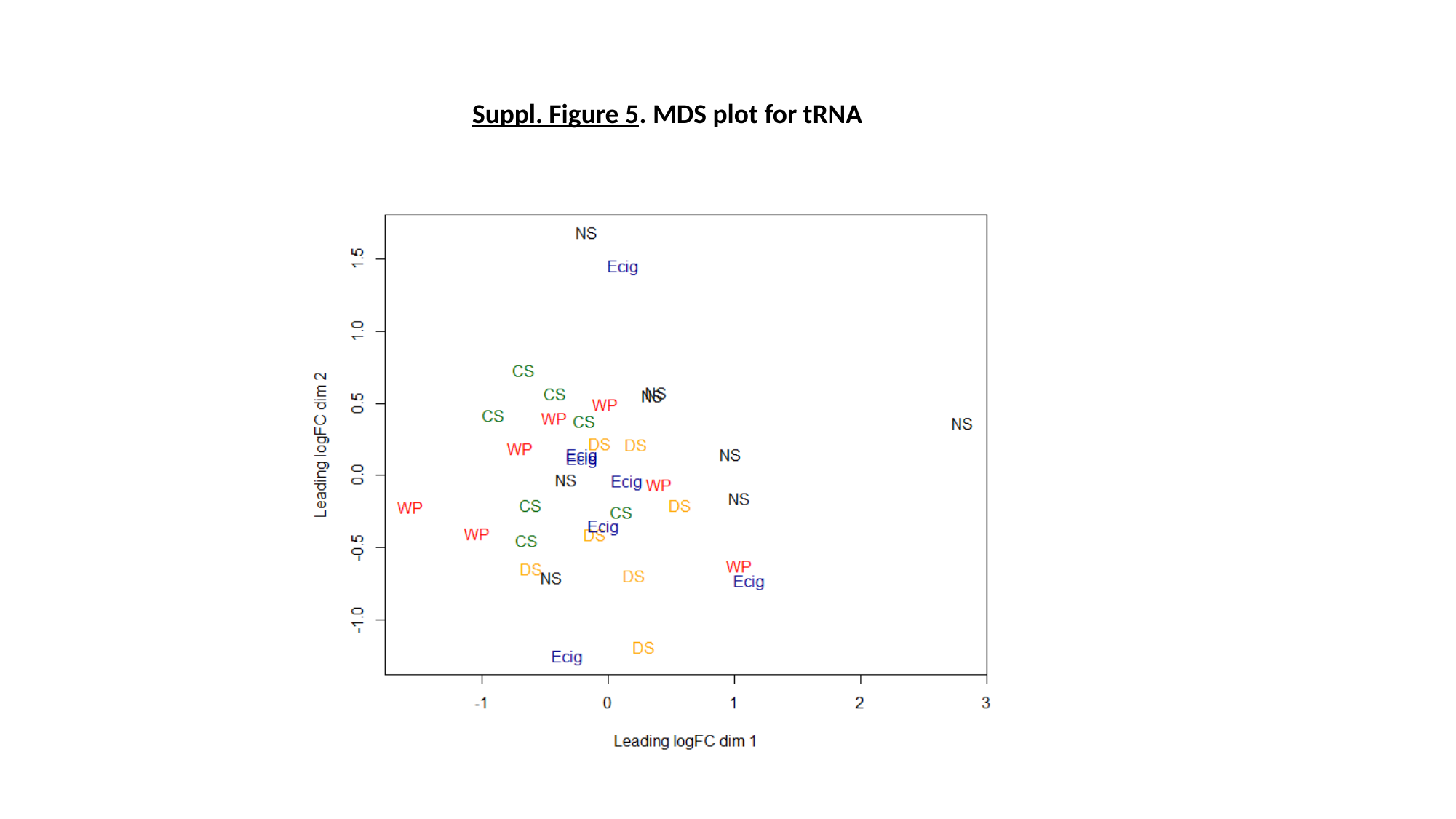

Suppl. Figure 5. MDS plot for tRNA

Supplement: Supplementary file 22 — Additional file 22: Supplementary Figure 5. Multidimensional Scaling based on differential tRNA expression in individual samples of non-smokers, cigarette smokers, waterpipe smokers, E-cig users and dual smokers. [file 12920_2020_748_MOESM22_ESM.pptx]

## Slide 1
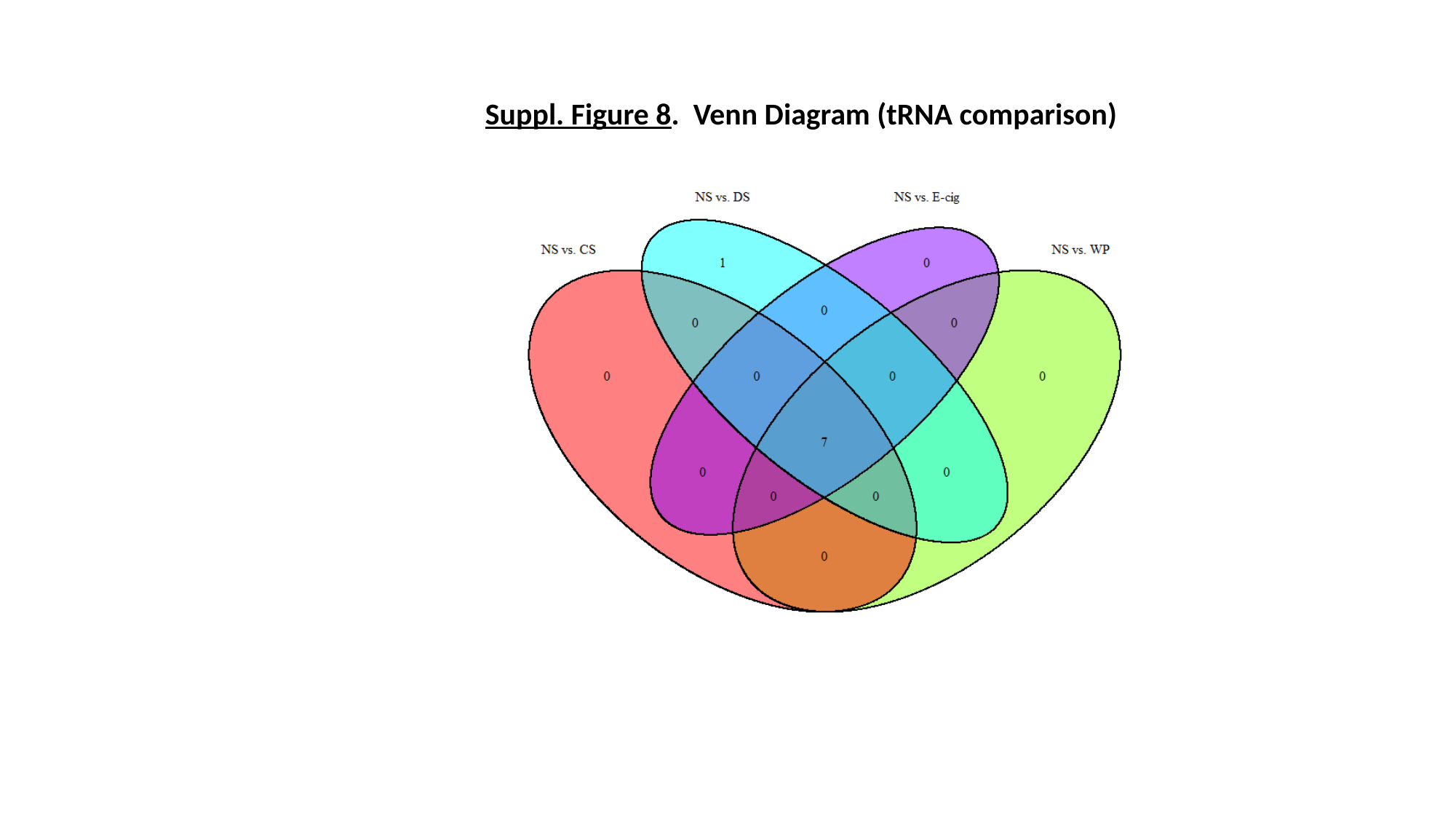

Suppl. Figure 8. Venn Diagram (tRNA comparison)

Supplement: Supplementary file 25 — Additional file 25: Supplementary Figure 8. Venn diagram showing the overlap of differentially expressed tRNAs identified from comparing four groups: non-smokers vs. cigarette smokers, non-smokers vs. waterpie smokers, non-smokers vs. E-cig users and non-smokers vs. dual smokers. [file 12920_2020_748_MOESM25_ESM.pptx]

## Slide 1
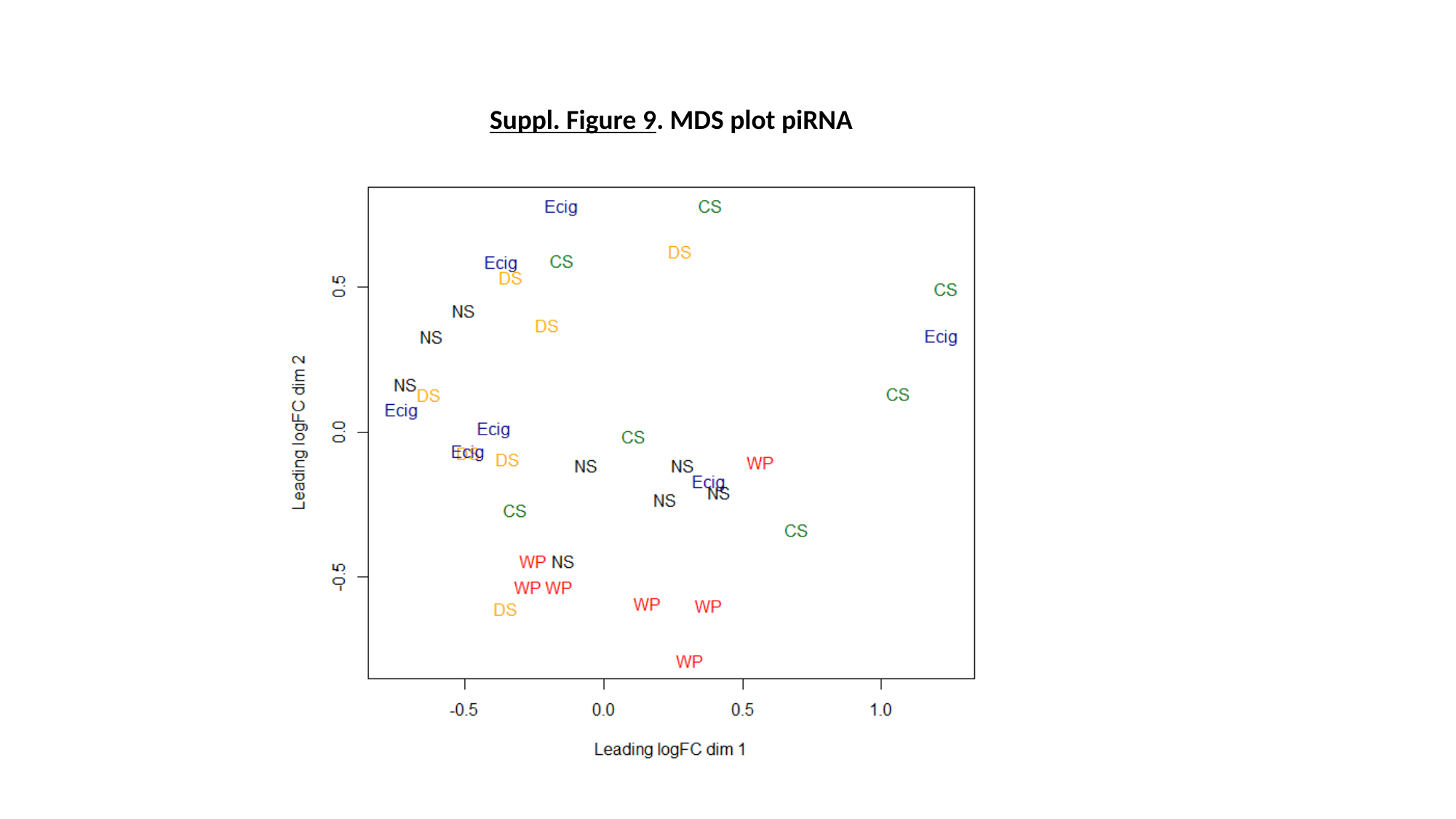

Suppl. Figure 9. MDS plot piRNA

Supplement: Supplementary file 26 — Additional file 26: Supplementary Figure 9. Multidimensional Scaling based on differential piRNA expression in individual samples of non-smokers, cigarette smokers, waterpipe smokers, E-cig users and dual smokers. [file 12920_2020_748_MOESM26_ESM.pptx]

## Slide 1
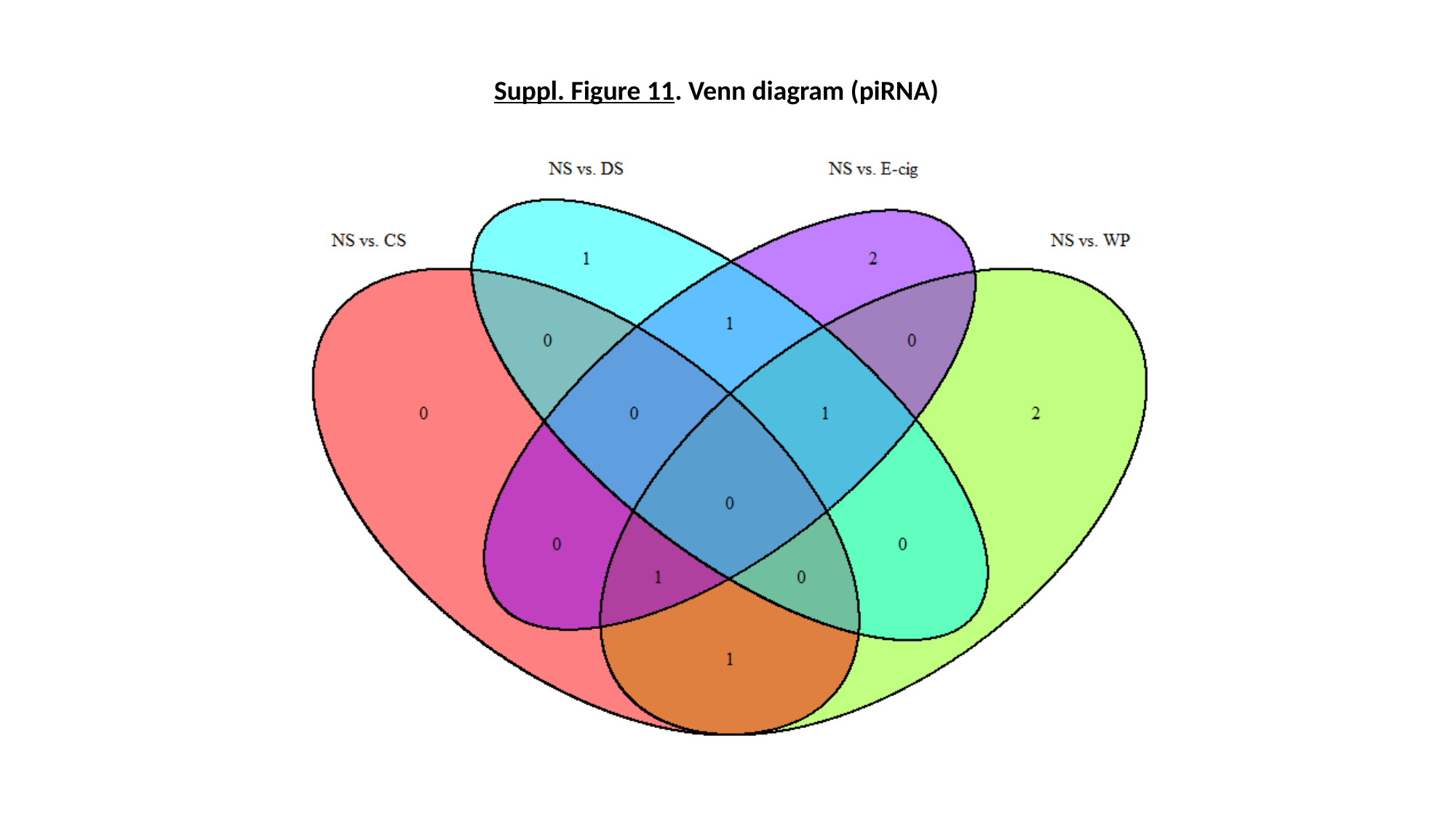

Suppl. Figure 11. Venn diagram (piRNA)

Supplement: Supplementary file 28 — Additional file 28: Supplementary Figure 11. Venn diagram showing the overlap of differentially expressed piRNAs identified from comparing four groups: non-smokers vs. cigarette smokers, non-smokers vs. waterpipe smokers, non-smokers vs. E-cig users and non-smokers vs. dual smokers. [file 12920_2020_748_MOESM28_ESM.pptx]
