# Supplementary material for: Exosomal microRNAs are novel circulating biomarkers in cigarette, waterpipe smokers, E-cigarette users and dual smokers
Source: BMC Med Genomics. 2020 Sep 10;13:128. doi: 10.1186/s12920-020-00748-3 (PMC7488025; doi:10.1186/s12920-020-00748-3)
Supplement: Supplementary file 23 — Additional file 23: Supplementary Figure 6. Volcano plot showing the relation between P-values of the changes in differentially expressed tRNA, and fold change in non-smokers, cigarette, waterpipe, E-cigarette users and dual smokers. (A) Non-smokers versus cigarette smokers. (B) Non-smokers versus waterpipe smokers. (C) Non-smokers versus E-cig users. (D) Non-smokers versus dual smokers. [file 12920_2020_748_MOESM23_ESM.pptx]

## Slide 1
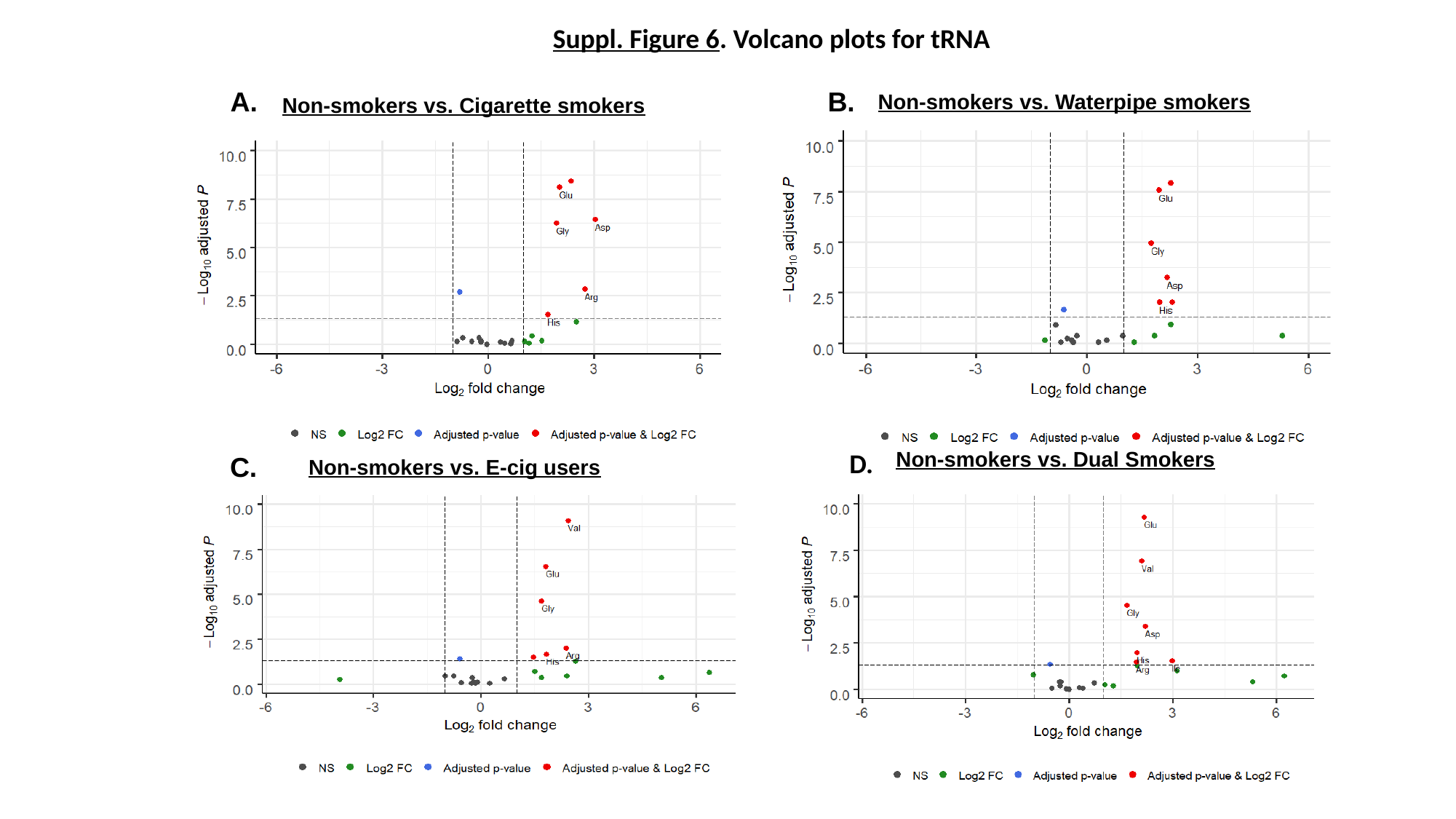

Suppl. Figure 6. Volcano plots for tRNA
A.
B.
Non-smokers vs. Waterpipe smokers
Non-smokers vs. Cigarette smokers
D.
Non-smokers vs. Dual Smokers
C.
Non-smokers vs. E-cig users
