# Supplementary material for: Exosomal microRNAs are novel circulating biomarkers in cigarette, waterpipe smokers, E-cigarette users and dual smokers
Source: BMC Med Genomics. 2020 Sep 10;13:128. doi: 10.1186/s12920-020-00748-3 (PMC7488025; doi:10.1186/s12920-020-00748-3)
Supplement: Supplementary file 24 — Additional file 24: Supplementary Figure 7. Hierarchical cluster analysis of differentially expressed miRNAs. (A) Heatmap clustering of the differentially expressed tRNAs significant among non-smokers vs. cigarette, smokers. (B) Heatmap clustering of the differentially expressed tRNAs significant among non-smokers vs. waterpipe smokers. (C) Heatmap clustering of the differentially expressed tRNAs significant among non-smokers vs. E-cigarette users. (D) Heatmap clustering of the differentially expressed tRNAs significant among non-smokers vs. dual smokers. These tRNAs were identified based on individual pairwise comparisons (with unadjusted raw p-value; P < 0.05). The analysis was generated using Z scores of the most differentially expressed significant tRNAs. The dendrogram shows clustering of pairwise comparisons among the different groups (non-smokers vs. cigarette smokers, non-smokers vs. waterpipe smokers, non-smokers vs. E-cigarette users and non-smokers vs. dual smokers). [file 12920_2020_748_MOESM24_ESM.pptx]

## Slide 1
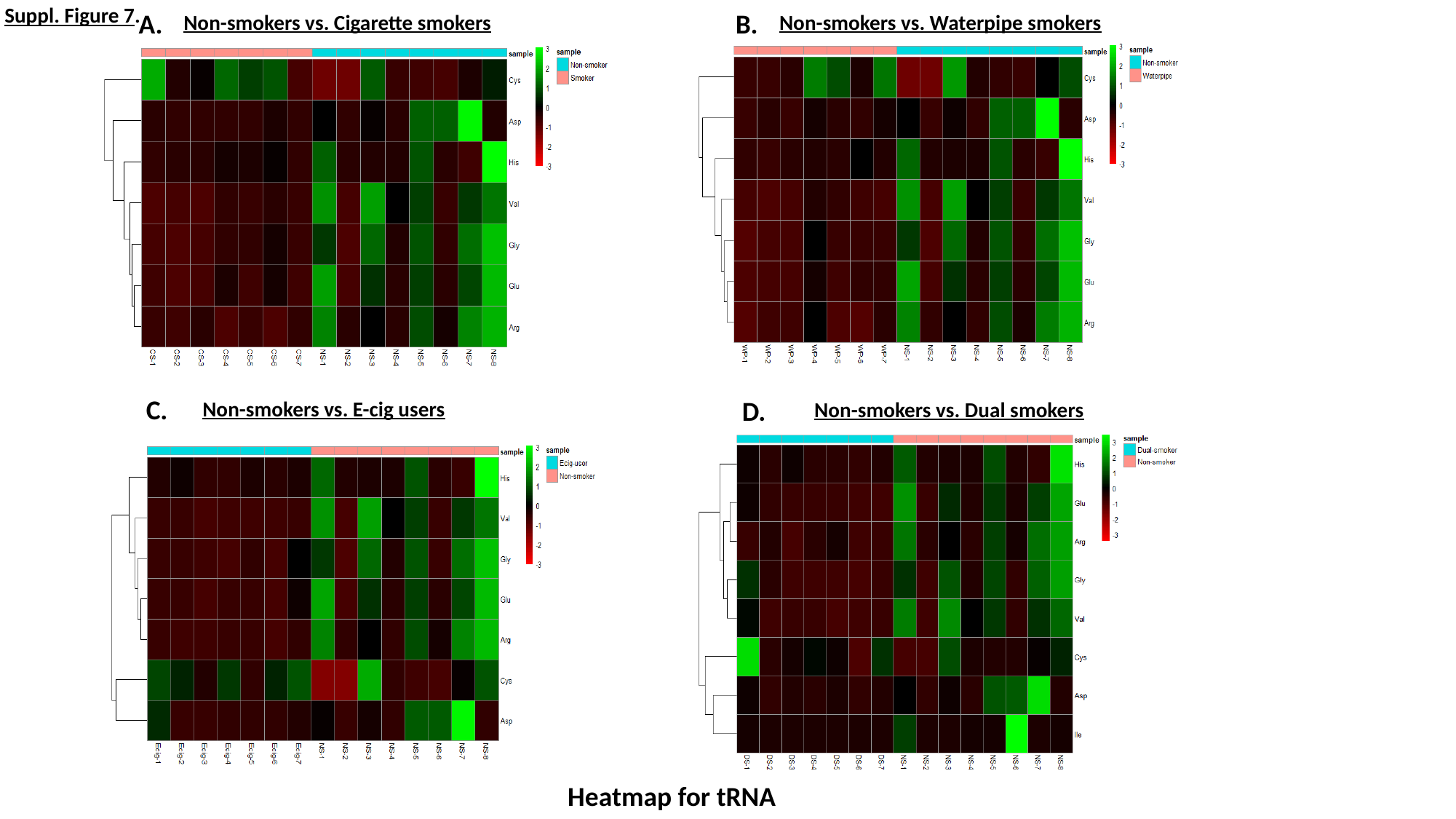

Suppl. Figure 7.
A.
B.
Non-smokers vs. Cigarette smokers
Non-smokers vs. Waterpipe smokers
C.
D.
Non-smokers vs. E-cig users
Non-smokers vs. Dual smokers
Heatmap for tRNA
