# Supplementary material for: Exosomal microRNAs are novel circulating biomarkers in cigarette, waterpipe smokers, E-cigarette users and dual smokers
Source: BMC Med Genomics. 2020 Sep 10;13:128. doi: 10.1186/s12920-020-00748-3 (PMC7488025; doi:10.1186/s12920-020-00748-3)
Supplement: Supplementary file 27 — Additional file 27: Supplementary Figure 10. Hierarchical cluster analysis of differentially expressed piRNAs. Heatmap clustering of the differentially expressed piRNAs significant among (A) non-smokers vs. cigarette smokers, (B) non-smokers vs. waterpipe smokers, (C) non-smokers vs. E-cigarette users, (D) non-smokers vs. dual smokers (E) cigarette smokers vs. waterpipe smokers, (F) cigarette smokers vs. E-cigarette smokers, (G) cigarette smokers vs. dual smokers, and (H) dual smokers vs. waterpipe smokers. These piRNAs were identified based on individual pairwise comparisons (with unadjusted raw p-value; P < 0.05). The analysis was generated using Z scores of the most differentially expressed significant piRNAs. The dendrogram shows clustering of pairwise comparisons among the different groups (non-smokers vs. cigarette smokers, non-smokers vs. waterpipe smokers, non-smokers vs. E-cigarette users and non-smokers vs. dual smokers). [file 12920_2020_748_MOESM27_ESM.pptx]

## Slide 1
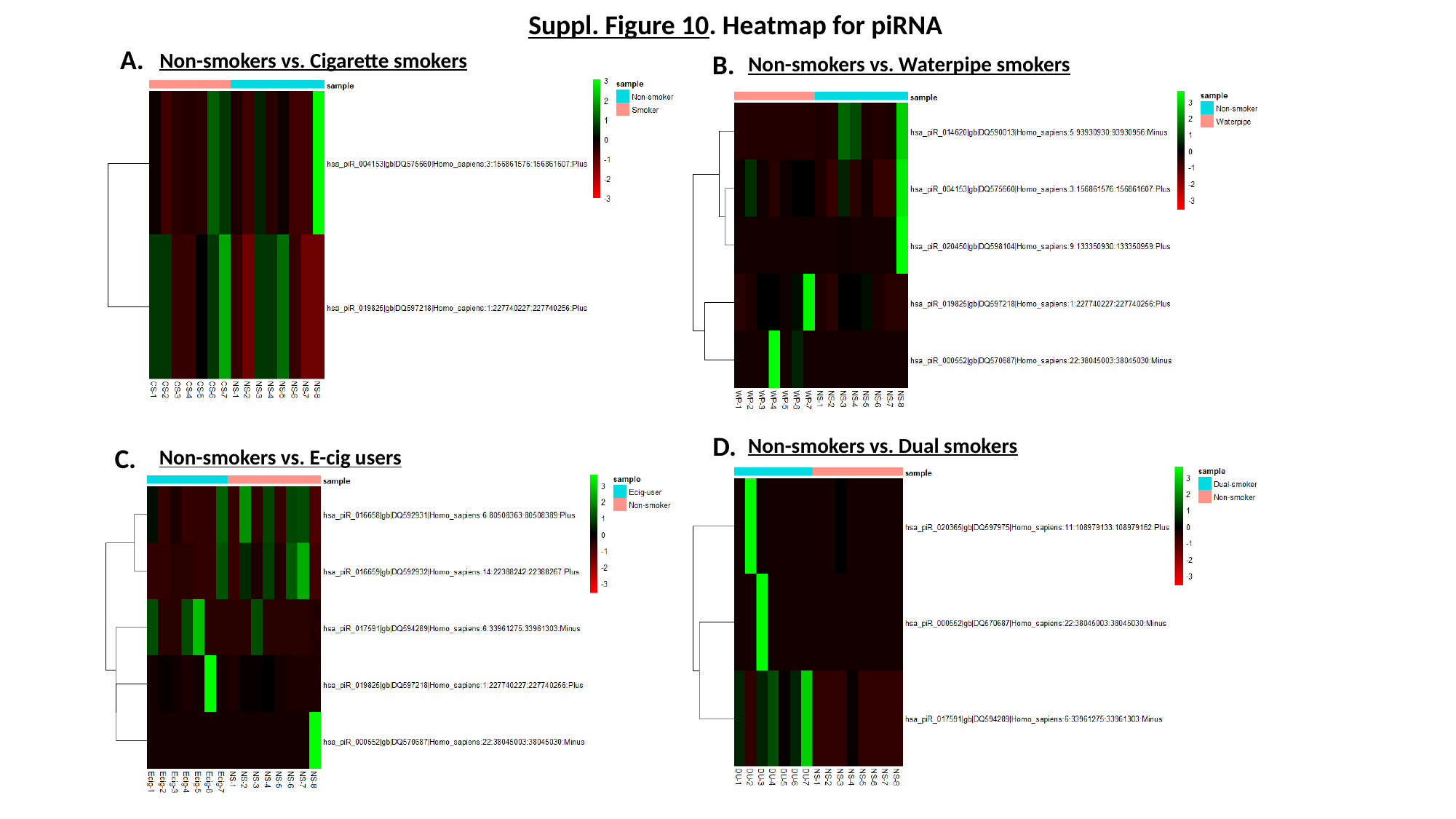

Suppl. Figure 10. Heatmap for piRNA
A.
Non-smokers vs. Cigarette smokers
B.
Non-smokers vs. Waterpipe smokers
D.
Non-smokers vs. Dual smokers
C.
Non-smokers vs. E-cig users

## Slide 2
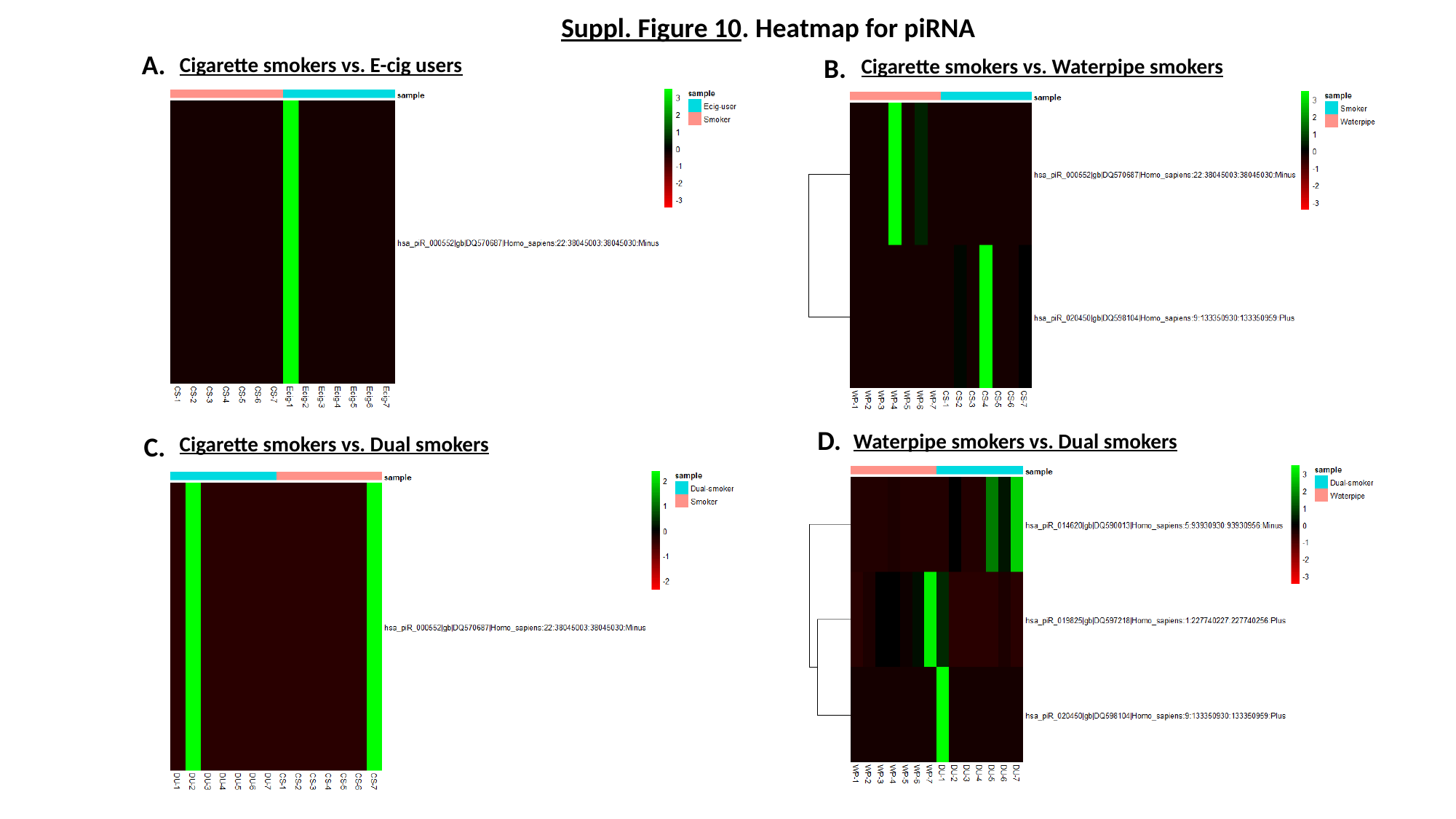

Suppl. Figure 10. Heatmap for piRNA
A.
B.
Cigarette smokers vs. E-cig users
Cigarette smokers vs. Waterpipe smokers
D.
Waterpipe smokers vs. Dual smokers
C.
Cigarette smokers vs. Dual smokers
